# Identification of Phytochemicals from Arabian Peninsula Medicinal Plants as Strong Binders to SARS-CoV-2 Proteases (3CL^Pro^ and PL^Pro^) by Molecular Docking and Dynamic Simulation Studies

**DOI:** 10.3390/molecules29050998

**Published:** 2024-02-25

**Authors:** Quaiser Saquib, Ahmed H. Bakheit, Sarfaraz Ahmed, Sabiha M. Ansari, Abdullah M. Al-Salem, Abdulaziz A. Al-Khedhairy

**Affiliations:** 1 Zoology Department, College of Sciences, King Saud University, P.O. Box 2455, Riyadh 11451, Saudi Arabia; alsalem1985@hotmail.com (A.M.A.-S.); kedhairy@ksu.edu.sa (A.A.A.-K.); 2 Department of Pharmaceutical Chemistry, College of Pharmacy, King Saud University, P.O. Box 2457, Riyadh 11451, Saudi Arabia; abakheit@ksu.edu.sa; 3 Department of Pharmacognosy, College of Pharmacy, King Saud University, P.O. Box 2457, Riyadh 11451, Saudi Arabia; sarahmed@ksu.edu.sa; 4 Botany & Microbiology Department, College of Sciences, King Saud University, P.O. Box 2455, Riyadh 11451, Saudi Arabia; sabiha.mahmood003@gmail.com

**Keywords:** SARS-CoV-2, 3CL^Pro^, PL^Pro^, medicinal plants, docking, molecular dynamic simulations

## Abstract

We provide promising computational (in silico) data on phytochemicals (compounds **1**–**10**) from Arabian Peninsula medicinal plants as strong binders, targeting 3-chymotrypsin-like protease (3CL^Pro^) and papain-like proteases (PL^Pro^) of SARS-CoV-2. Compounds **1**–**10** followed the Lipinski rules of five (RO5) and ADMET analysis, exhibiting drug-like characters. Non-covalent (reversible) docking of compounds **1**–**10** demonstrated their binding with the catalytic dyad (CYS145 and HIS41) of 3CL^Pro^ and catalytic triad (CYS111, HIS272, and ASP286) of PL^Pro^. Moreover, the implementation of the covalent (irreversible) docking protocol revealed that only compounds **7**, **8**, and **9** possess covalent warheads, which allowed the formation of the covalent bond with the catalytic dyad (CYS145) in 3CL^Pro^ and the catalytic triad (CYS111) in PL^Pro^. Root-mean-square deviation (RMSD), root-mean-square fluctuation (RMSF), and radius of gyration (Rg) analysis from molecular dynamic (MD) simulations revealed that complexation between ligands (compounds **7**, **8**, and **9**) and 3CL^Pro^ and PL^Pro^ was stable, and there was less deviation of ligands. Overall, the in silico data on the inherent properties of the above phytochemicals unravel the fact that they can act as reversible inhibitors for 3CL^Pro^ and PL^Pro^. Moreover, compounds **7**, **8**, and **9** also showed their novel properties to inhibit dual targets by irreversible inhibition, indicating their effectiveness for possibly developing future drugs against SARS-CoV-2. Nonetheless, to confirm the theoretical findings here, the effectiveness of the above compounds as inhibitors of 3CL^Pro^ and PL^Pro^ warrants future investigations using suitable in vitro and in vivo tests.

## 1. Introduction

SARS-CoV-2, a causative agent for the COVID-19 disease, was first reported in 2019 from Wuhan City, China. As of 22 November 2023, the World Health Organization (WHO) has officially listed 772,166,517 confirmed cases of COVID-19 and 6,981,263 deaths globally [1]. This crushing pandemic is still affecting the national healthcare system as well as disrupting the global economy. Though several theories have been linked to the emergence of SARS-CoV-2, the source of SARS-CoV-2 is still unidentified. Humans infected with SARS-CoV-2 typically show symptoms of fever, dry cough, shortness of breath, dyspnea, headache, fatigue, diarrhea, and bilateral lung infiltrates. Being highly transmissible among humans, SARS-CoV-2 has a median incubation period of 4 days, with the longest being 41 days [2,3]. COVID-19 patients also showed disease progression to pneumonia by acute respiratory distress syndrome (ARDS), which leads to septic shock and ultimately causes death, chiefly through cytokine storm [4,5]. 

SARS-CoV-2 belongs to the β genus of the Coronaviridae family, which is typically characterized as enveloped viruses [6]. SARS-CoV-2 contains a positive-sense single stranded RNA (~30 kb) as its nucleic acid. There are 14 open reading frames (ORFs) in SARS-CoV-2. Together, they encode for 27 different proteins, including the major four proteins, i.e., the nucleocapsid (N), spike (S), envelope (E), and membrane (M) proteins [7,8]. Transcription of the SARS-CoV-2 genome generates large polyproteins (~800 kDa), which are proteolytically cleaved to generate non-structural proteins (NSPs) that are essential for viral replication [9,10]. The cleavage of this long polypeptide is governed by a specific proteolytic enzyme known as main protease (M^Pro^) or 3CL^Pro^ and papain-like protease (PL^Pro^) to release sixteen NSPs [11]. Despite the high frequency of mutation in SARS-CoV-2, the sequences of 3CL^Pro^ and PL^Pro^ are highly conserved owing to the fact that mutations of such indispensable proteins are often deadly to the virus [11,12,13]. Viewing the crucial role of 3CL^Pro^ and PL^Pro^ in viral replication, growth, and survival, they have been recognized as potential targets for the development of novel drugs against SARS-CoV-2. 

There is 96% amino acid sequence similarity and conservation of active residues of the 3CL^Pro^ protein between SARS-CoV and SARS-CoV-2. In this connection, a study conducted with plant extracts and natural compounds derived from mushroom exhibited a replication block of SARS-CoV by inhibiting 3CL^Pro^, indicating it as a potent target for SARS-CoV-2 inhibition [14]. Other classes of natural compounds that have inhibited 3CL^Pro^ of SARS-CoV include flavonoids, lignoid, terpenoid, tanshinone, chalcone, diarylheptanoid, biphenyl propanoids [15]. Hence, the small molecules from natural sources having the potential to inhibit 3CL^Pro^ of SARS-CoV could also inhibit 3CL^Pro^ of SARS-CoV-2 [16]. In the same line, there is an 83% similarity between the active amino acid residues and protein sequences that exist between SARS-CoV and SARS-CoV-2 PL^Pro^. Consequently, the compounds that have been responsible for the inhibition of PL^Pro^ in SARS-CoV may also inhibit and be effective against SARS-CoV-2 PL^Pro^ [16]. The known inhibitors of PL^Pro^ are derived from natural sources and belong to the class of flavonoids: chalcone, tanshinone, polyphenol, diarylheptanoid, biphenyl propanoids, and cinnamic amide [15]. Very few research studies have been conducted either in vitro or in vivo on the efficacy of varying nutraceuticals or naturally derived compounds to analyze their inhibition properties against SARS-CoV-2. 

However, a traditional Chinese medicine called Pudilan Xiaoyan Oral Liquid, made from four herbs and >180 ingredients, demonstrated its potent efficacy as anti-SARSCoV-2 in infected hACE2 mice [17]. In the same line, during the outbreak of COVID-19 in Wuhan, patients treated with Chinese traditional Shuanghuanglian oral liquid (SHL) in combination with Western drugs exhibited significant changes in health effects [18]. Also, a nebulized formula of quercetin and N-acetylcysteine significantly relieved the respiratory symptoms of COVID-19 patients treated with hydroxychloroquine and antibiotics [19]. In China, >90% of COVID-19 patients have received traditional Chinese medicine, which has exhibited an effective rate exceeding 90% [20]. A novel traditional Chinese medicine formula, Taiwan Chingguan Yihau (NRICM101), was administered to 33 patients with COVID-19. They showed three consecutive negative results for the infection, with a median of nine days. Pharmacological evaluation of the drug exhibited that NRICM101 inhibited ACE2, 3CL^Pro^, and plaque formation, and reduced the cytokine storm (IL-6 and TNF-α) [21]. In the enzymatic assay, Jingfang Granules extract demonstrated inhibitory effects against 3CL^Pro^ and PL^Pro^ [22]. In the same line, several plant extracts, including turmeric, mustard, and wall rocket, significantly inhibited the activity of 3CL^Pro^ [23]. Root chicory extract demonstrated anti-SARS-CoV-2 effects through inhibitory effects against both proteases (3CL^Pro^ and PL^Pro^) [24]. Curcumin affected the replicative cycle and demonstrated virucidal effects on SARS-CoV-2 [25]. Moreover, quercetin and its synthetic analogues have been regarded as promising agents to inhibit 3CL^Pro^ and PL^Pro^ [26,27,28].

In spite of the promising results from the repurposed drugs and herbal medicines, it is essential to examine their mechanism of inhibition, safety, and efficacy for their prospective application in the COVID-19 treatment. Consequently, computational approaches are adopted to search for suitable drugs, especially when the biological hazards are associated with SARS-CoV-2. In this context, computational methods, including molecular docking, molecular dynamic (MD) simulations, and in silico ADMET, are adopted to find out the potential drugs, phytochemicals, and purified compounds [29,30,31,32]. Understanding this fact, most of the computational studies have either focused on downloading the list of phytochemicals from the public database or drawing the structures using the software and analyzing them for their drug-like efficacy using computational programming. Such approaches not only created a data gap, but valuable information on the efficacy of phytochemicals is still missing, especially their effectiveness as a drug in different test models. Hence, we judiciously selected ten phytochemicals (compounds **1**–**10**) from Arabian Peninsula medicinal plants that our research group had previously isolated and purified, and whose structures we had characterized. In addition, the selected compounds (**1**–**10**) have already been evaluated by our research group for their potential as drugs against the hepatitis B virus, antidiabetic, anti-inflammatory, and cytotoxicity properties in different test models [33,34,35]. Hence, relying on their drug-like characteristics, we have adopted in silico (bioinformatic) approaches to evaluate the potential of compounds **1**–**10** to inhibit dual targets (3CL^Pro^ and PL^Pro^) of SARS-CoV-2. Consequently, compounds **1**–**10** were assessed for their drug-likeness characteristics by analyzing their ADMET properties, molecular docking (non-covalent and covalent) interactions with 3CL^Pro^ and PL^Pro^, and MD simulations (RMSD, RMSF, and Rg) to quantify ligand–protein conformational stability and the compactness of amino acids after interactions.

## 2. Results

### 2.1. Drug-Likeness and ADMET Analysis of Compounds **1**–**10**

Details of compounds **1**–**10** and their 2D structures are listed in Table 1. The drug-like characteristics of compounds **1**–**10** were determined by studying their physicochemical properties. Compounds **1**, **4**, **5**, **6**, **7**, **8**, and **9** showed drug-likeness characters by qualifying both the Veber and RO5 rules (Table 2). Compounds **4** and **5** followed all the parameters of RO5, except violating only one criterion of lipophilicity (LogP > 5). On the other hand, compounds **2**, **3**, and **10** violated more than two parameters. 

**Figure 1:**
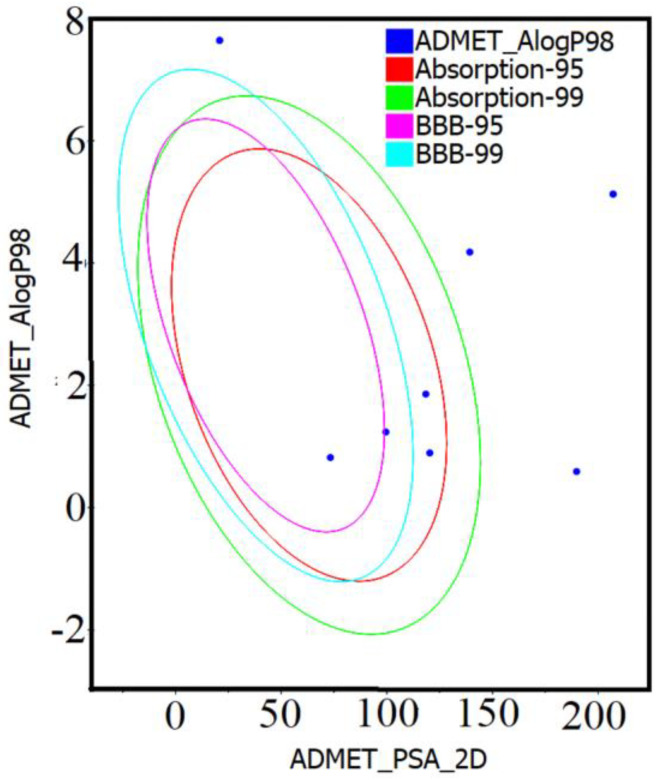
AlogP98 versus 2D PSA ellipses plot. AlogP98 versus 2D PSA ellipses plotted from the calculated values of ADMET indicate the confidence levels (95% and 99%) of HIA and BBB penetration models.

Compounds **1**–**10** were further analyzed for their in silico predictions about their properties of absorption, distribution, metabolism, excretion, and toxicity (ADMET) behavior. Compounds **6**, **7**, **8**, and **9** showed good human intestinal absorption (HIA) by meeting the criteria of the above rules. However, the HIA of compound **1** was low, whereas the HIA of the remaining compounds (**2**, **3**, **4**, **5**, **10**) was extremely low (Table 3). All compounds were further analyzed by the ADMET solubility descriptor, chiefly relying on the molar solubility of drugs within different scales (0 to 5) or log (Sw) < −8.0 to 0.0. Following this scale, compound **1** showed aqueous solubility, but it was low-soluble. On the other hand, the solubility of compounds **2**, **3**, **4**, and **5** was found to be very low, but it was possible to make them soluble in an aqueous solution. Interestingly, compounds **6**, **7**, **8**, **9**, and **10** showed good solubility in the aqueous solution (Table 3). The predicted data for ADMET showed that compounds **1**–**10** are non-hepatotoxic. Compounds **4, 5, 7**–**9** showed >90% of plasma protein binding (PPB), while compounds **1**–**3**, **6,** and **10** exhibited <90% of PPB. On the other hand, blood–brain barrier partition (BBBP) levels for compounds **1**–**6**, **8** and **10** were very low, while those for compounds **7** and **9** had a low BBBP level. Compounds **5** and **7**–**10** exhibited non-inhibition of the enzyme CYP2D6, while compounds **1**–**4** and **6** showed inhibition. Compounds **6**–**9** meet the criteria of AlogP98 (<5) and PSA_2D (<140 Å2) for optimal cell permeability (Table 3, Figure 1).

### 2.2. Explanation of Active Sites in 3CL^Pro^ and PL^Pro^

**Figure 2:**
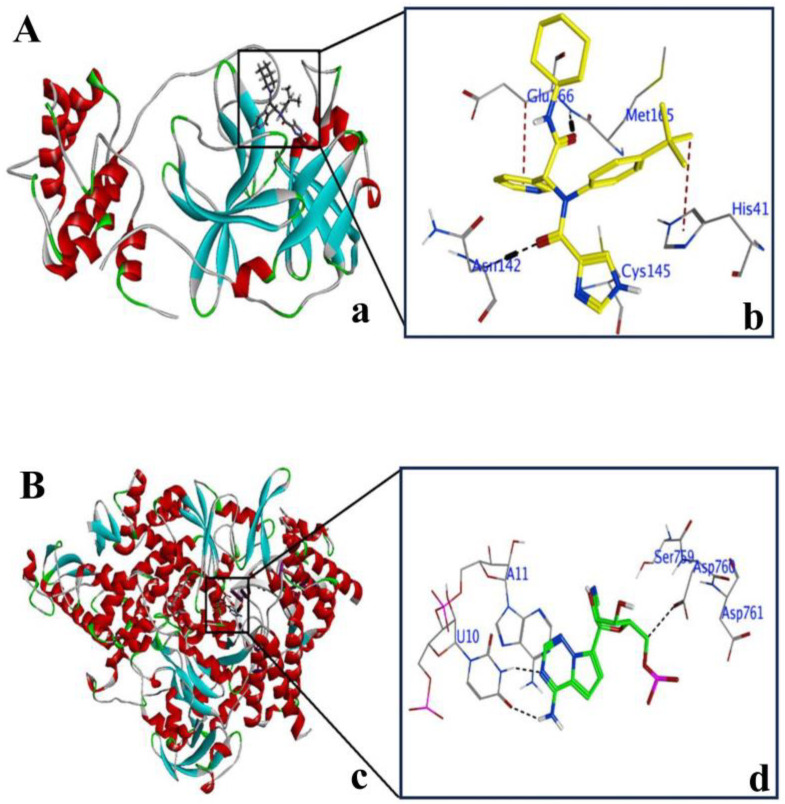
Active site in SARS-CoV-2 target proteins 3CL^Pro^ and PL^Pro^. (**Aa**) Ribbon structure of 3CL^Pro^ (PDB ID: 6W63) with ligand (X77) in the active site of the target protein. (**Ab**) Magnified view of the active site of 3CL^Pro^ showing the catalytic dyad of residues interacting with X77. (**Bc**) Ribbon structure of PL^Pro^ (PDB ID: 6WUU) with ligand (VIR250) in the active site of the target protein. (**Bd**) Magnified view of the active site of PL^Pro^ showing the catalytic triad of residues interacting with VIR250. Helices are red, beta sheets are cyan, turns are green, and coils are white. Images are generated by using MOE software (version 2015.1).

Prior to the commencement of docking studies of compounds **1**–**10** with 3CL^Pro^ and PL^Pro^, we first re-confirmed the active sites in the above target proteins, which were reported earlier [36,37]. The active site in 3CL^Pro^ contains crucial residues (i.e., CYS145 and HIS41) in the catalytic dyad. 3CL^Pro^ contains other important residues, including CYS145, HIS41, GLY143, SER144, THR26, THR24, and LEU27, as also reported in a previous study [38] (Figure 2(Aa,b)). PL^Pro^ has also been reported to contain its active site at the interface between the thumb and palm subdomains [39]. Within this active site, CYS111, HIS272, and ASP286 residues have been found as a catalytic triad. In addition, TYR268, and GLN269 have been recognized as important residues in the PL^Pro^ active site (Figure 2(Bc,b)).

### 2.3. Validation of Docking Protocol with 3CL^Pro^ and PL^Pro^

We further performed the validation of the docking protocol by redocking the co-crystal ligand (X77) with 3CL^Pro^, which showed hydrogen–donor (H-bond), hydrogen–acceptor (H-acceptor), H-pi (H-π) and pi-H (π-H) interactions. At a distance of 4.07 Å and 3.03 Å, H–donor interactions occurred between the C17 (numbering of atoms generated by MOE software) atom of ligand and MET49 residue, N29 atom of ligand and HOH671 residue in 3CL^Pro^. At a distance of 3.01, 2.94, and 2.8 Å, the O33, O33, and O34 atoms of the ligand showed H–acceptor interactions with ASN142, GLY143, and GLU166 residues in 3CL^Pro^. H-π interaction developed at a distance of 4.31 Å between the C21 atom of the ligand and the HIS41 residue of 3CL^Pro^. Also, the 6-ring of the ligand showed π-H interaction with the GLU166 residue in 3CL^Pro^ at a distance of 3.8 Å (Supplementary Table S1). The superimposed co-crystalized ligand docked in the same position in the active site of the crystalized ligand (X77) in 3CL^Pro^ (Supplementary Figure S1). The RMSD of the co-crystalized ligand superimposed with the crystalized ligand was found to be 0.557 Å for 3CL^Pro^. The RMSD value from the redocking analysis was below the qualifying criteria of <3 Å. Furthermore, the reliability of the docking protocol was performed by redocking the co-crystal ligand (VIR250) with PL^Pro^. Redocking resulted in the development of H-donor and H-acceptor interactions. H-donor interactions developed at a distance of 3.09, 3.2, and 3.07 Å between N32, CB38, and OE2 65 atoms of ligand and ASP164, ASP164, and CYS111 residues in PL^Pro^. At a distance of 2.98 Å, H-acceptor binding developed between the O2 atom of the ligand and the ARG166 residue in PL^Pro^ (Supplementary Table S2). The superimposed co-crystalized ligand also docked in the same position in the active site of crystalized VIR250 in PL^Pro^ (Supplementary Figure S2). The RMSD of co-crystalized ligand superimposed with the crystalized ligand was 2.927 Å for PL^Pro^, which was <3 Å. 

### 2.4. Non-Covalent Docking of Compounds **1**–**10** with 3CL^Pro^

**Figure 3:**
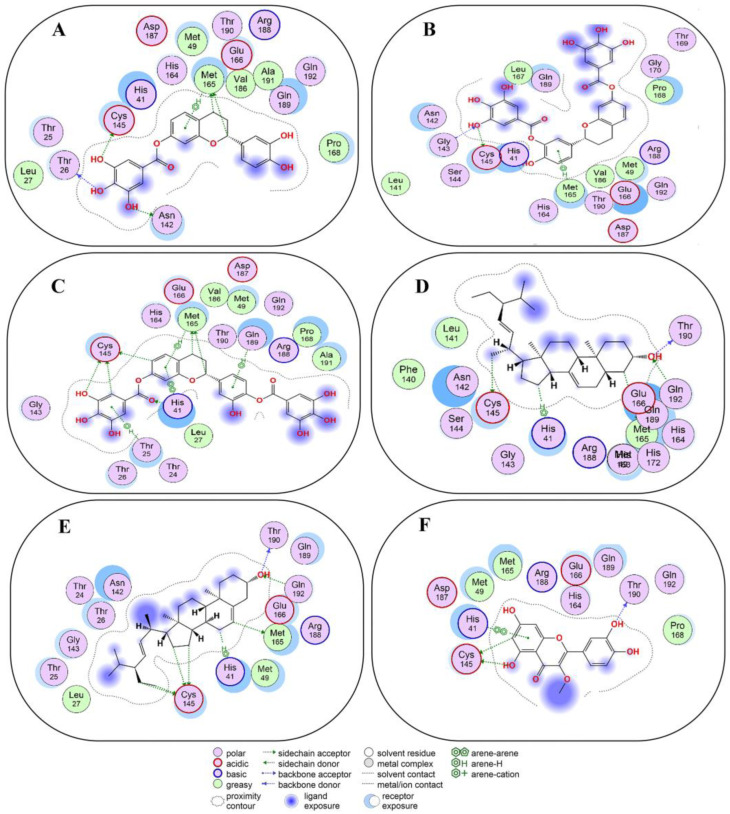
Two-dimensional view of 3CL^Pro^ showing non-covalent binding with compound **1** (**A**), compound **2** (**B**), compound **3** (**C**), compound **4** (**D**), compound **5** (**E**), and compound **6** (**F**). The surface representation and magnified view of compounds **1**–**6** in the active site of 3CL^Pro^ is shown in Supplementary Figures S3–S8. Images are generated by using MOE software.

Post validation of the docking protocol, compounds **1**–**10**, as strong binders, were analyzed for non-covalent docking with 3CL^Pro^. 5,3′,4′-trihydroxyflavan 7-O-gallate (Compound **1**) occupied the active site and exhibited favorable H–donor and π-H interactions with the residues of 3CL^Pro^ (Figure 3A and Supplementary Figure S3A,B). H–donor interaction was found at a distance of 4.03 and 3.55 Å between the C12 and C17 atoms of compound **1** and the MET165 residue in 3CL^Pro^. Also, O31, O34, and O37 atoms of compound **1** showed H–donor interactions with ASN142, THR26, and CYS145 residues of 3CL^Pro^ at a distance of 2.97, 3.25, and 3.75 Å. π-H interaction was found at a distance of 3.72 Å between the 6-ring of compound **1** and the MET165 residue of 3CL^Pro^ (Supplementary Table S3).

5,4′-dihydroxyflavan 7-3′-O-digallate (compound **2**) and 3CL^Pro^ exhibited active site binding by means of H–donor, H–acceptor, and π-H interactions with different residues in the active site of the target protein (Figure 3B and Supplementary Figure S4A,B). The O58 atom of compound **2** showed H–donor interaction with residue CYS145 at a distance of 3.27 Å and H–acceptor interaction with residue GLY143 at a distance of 3.05 Å. While the 6-ring of compound **2** exhibited π-H interaction with the MET165 residue of 3CL^Pro^. Compound **2** showed a docking score of −7.4 kcal/mol, which was near the score of −8.4 kcal/mol found with co-crystalized ligand (X77) with 3CL^Pro^ (Supplementary Table S4).

5,3′-dihydroxyflavan 7-4′-O-digallate (compound **3**) also occupied the active site of 3CL^Pro^ (Figure 3C and Supplementary Figure S5A,B). Thereby, compound **3** interacted with the catalytic dyad as well as other residues. At the distances of 3.75, 4.38, and 3.58 Å, the C7, O10, and C19 atoms of compound **3** interacted as H-donors with the CYS145 residue of 3CL^Pro^. At a distance of 3.81 Å, the C30 atom of compound **3** interacted as H-donor with the MET165 residue of 3CL^Pro^. At a distance of 2.98 Å, the O16 atom of compound **3** interacted as H–acceptor with the HIS41 residue of 3CL^Pro^. π-H interactions were developed between the 6 ring of compound **3** and the THR25, MET165, and GLN189 residues of 3CL^Pro^. 

π-π interaction developed only between the HIS41 residue and the 6-ring of compound **3**. Also, compound **3** showed a docking score of -7.9 kcal/mol, which was near the −8.4 kcal/mol obtained from a co-crystalized ligand (X77) with 3CL^Pro^ (Supplementary Table S5).

Spinasterol (compound **4**) showed interactions within the active site of 3CL^Pro^ (Figure 3D and Supplementary Figure S6A,B). Compound **4**, at a distance of 2.74 Å and 4.2 Å exhibited H–donor interaction between O1 and THR190, C22, and MET165 residues of 3CL^Pro^. The C60 atom of compound **4**, at a distance of 4.06 Å showed H–donor interaction with one of the catalytic dyad residues (CYS145) of 3CL^Pro^. At a distance of 4.05 Å, the C27 atom of compound **4** exhibited H-π interaction with another catalytic dyad (HIS41) of 3CL^Pro^. H-acceptor interaction was found between the O1 atom and the GLN192 residue at a distance of 3.23 Å (Supplementary Table S6).

Stigmasterol (compound **5**) predominantly resided in the active site of 3CL^Pro^ and interacted with the residues there (Figure 3E and Supplementary Figure S7A,B). At distances of 3.92, 4.21, 4.06, and 3.77 Å, the C1, C5, C22, and C25 atoms of compound **5** exhibited H–donor interactions with the CYS145 residue (catalytic dyad). In addition, at a distance of 3.41 and 3.16 Å, C35 and O43 atoms of compound **5** also demonstrated H–donor interactions with MET165 and THR190 residues of 3CL^Pro^. On the other hand, at a distance of 4.21 Å, the C32 atom of compound **5** showed H-π interaction with another catalytic dyad (HIS41) of 3CL^Pro^. Similarly, at 3.16 Å, the O43 atom of compound **5** showed H–acceptor interaction with the GLN192 residue of 3CL^Pro^ (Supplementary Table S7). 

3′,4′,5,7-tetrahydroxy-3-methoxyflavone (compound **6**) resided in the active site and interacted with the catalytic dyad and other residues of 3CL^Pro^ (Figure 3F and Supplementary Figure S8A,B). The O27 and O29 atoms of compound **6** exhibited H–donor interactions with one of the catalytic dyads (CYS 145) at distances of 3.12 and 3.86 Å. Also, the O19 atom of compound **6** showed H–donor interaction at the distance of 2.8 Å with the THR190 residue of 3CL^Pro^. Moreover, at a distance of 3.63 Å, π-π stacking interaction developed between the 6-ring of compound **6** and HIS41, another catalytic dyad (Supplementary Table S8). 

**Figure 4:**
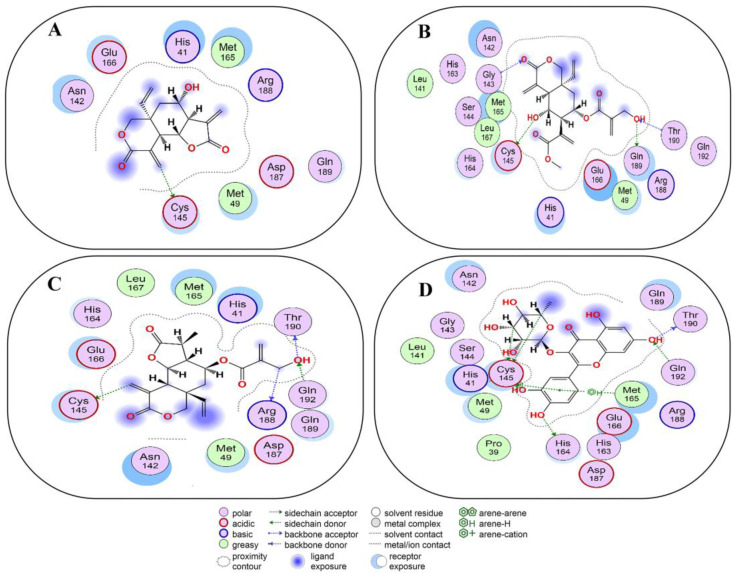
Two-dimensional view of 3CL^Pro^ showing non-covalent binding with compound **7** (**A**), compound **8** (**B**), compound **9** (**C**), and compound **10** (**D**). The surface representation and magnified view of compounds **7**–**10** in the active site of 3CL^Pro^ is shown in Supplementary Figures S9–S12. Images were generated by using MOE software.

Vernolepin (compound **7**) occupied the active site of the target protein 3CL^Pro^ and exhibited only H–donor interaction (Figure 4A and Supplementary Figure S9A,B). Compound **7,** the C14 atom, at a distance of 3.56 Å, interacted as an H–donor with one of the residues (CYS145) of the catalytic dyad in 3CL^Pro^ (Supplementary Table S9).

Vernadolol (compound **8**) also resided in the active site of 3CL^Pro^ (Figure 4B and Supplementary Figure S10A,B). Within the active site, compound **8** exhibited two types of interactions (H–donor and H–acceptor) with the residues. At a distance of 3.43 and 2.97 Å, the O21 and O48 atoms of compound **8** showed H–donor interaction with CYS145 and GLN189 residues. As H-acceptors, O12 and O48 atoms of compound **8**, at a distance of 3.41 and 3.5 Å, interacted with GLY143 and THR190 residues (Supplementary Table S10). 

11β,13-dihydrovernodalin (compound **9**) inhabited the active site and showed two types of interactions: H–donor and H–acceptor with 3CL^Pro^ residues (Figure 4C and Supplementary Figure S11A,B). O7 (2.85 Å), C21 (3.56 Å), and C25 (3.52 Å) atoms of compound **9** showed H–donor interactions with THR190, CYS145, and AGR188. At a distance of 3.34 Å, the O7 atom of compound **9** interacted as an H-acceptor by binding with the GLN192 residue of 3CL^Pro^ (Supplementary Table S11). 

Quercitrin 3-O-rhamnoside (compound **10**) occupied the active site of 3CL^Pro^ and showed binding with the catalytic dyad, as well as other residues of 3CL^Pro^ as H–donor, H–acceptor, π-H, and π-π interactions (Figure 4D and Supplementary Figure S12A,B). At a distance of 3.47, 2.88, and 4.49 Å, the C5, O37, and C45 atoms of compound **10** showed binding with one of the catalytic dyads (CYS145). While the 6-ring of compound **10** binds with another catalytic dyad (HIS41) at a distance of 3.71 Å by the formation of π-π stacking. Also, the 6-ring of compound **10** binds with MET165 at a distance of 4.06 Å via π-H interaction. THR190 and HIS164 residues of 3CL^Pro^ bind with the O21 and O34 atoms of compound **10** by H–donor interaction. At a distance of 3.17 Å, the O21 atom of compound **10** binds with the GLN192 residue via H-acceptor means. The docking score of compound **10** (−7.6 kcal/mol) was near the docking score (−8.4 kcal/mol) of X77 ligand co-crystalized with 3CL^Pro^ (Supplementary Table S12).

### 2.5. Non-Covalent Docking of Compounds **1**–**10** PL^Pro^

**Figure 5:**
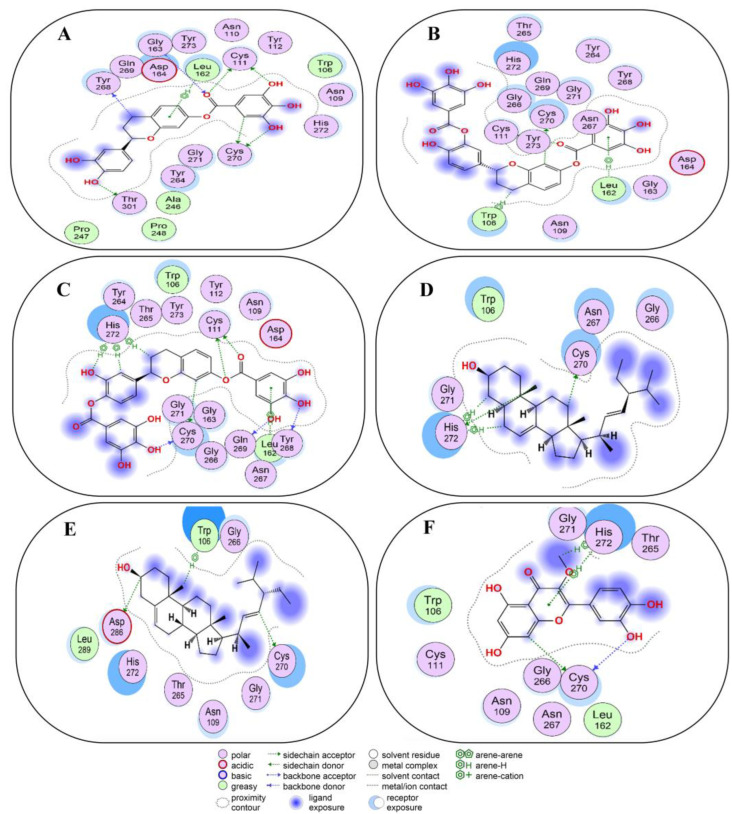
Two-dimensional view of PL^Pro^ showing non-covalent binding with compound **1** (**A**), compound **2** (**B**), compound **3** (**C**), compound **4** (**D**), compound **5** (**E**), and compound **6** (**F**). Surface representation and magnified view of compounds **1**–**6** in the active site of PL^Pro^ is shown in Supplementary Figures S13–S18. Images are generated by the use of MOE software.

The non-covalent docking of compounds was further evaluated for their binding potential with PL^Pro^. Compound **1** showed its occupancy in the active site of PL^Pro^ (Figure 5A and Supplementary Figure S13A,B). Compound **1** interacted with varying types of amino acid residues predominantly through H–donor activities as well as H–acceptor and π-H interactions. Specifically, the O41 (3.55 Å) atom of compound **1** acted as H-donor to bind with the catalytic triad (CYS111) in the active site of PL^Pro^. Other atoms of compound **1**, including O5 (3.04 Å), C17 (3.22 Å), C28 (3.78 Å), O31 (3.19 Å), and O37 (3.7 Å), interacted as H-donors with THR301, TYR268, and CYS270 amino acid residues. In addition, compound **1** also showed H–acceptor and π-H interactions with its O41 and 6-ring atoms with GLY163 (3.64 Å) and LEU162 (3.99 Å) residues in the target protein (Supplementary Table S13). 

Compound **2** docked into the active site of PL^Pro^ thereby showing H–donor, H-π, and π-H interactions with the amino acids (Figure 5B and Supplementary Figure S14A,B). As an H-donor, the O16 (2.88 Å) atom of compound **2** showed interaction with CYS111, an amino acid in the catalytic triad of PL^Pro^. Also, the C34 atom of compound **2** binds to the CYS270 residue at a distance of 3.71 Å. The C24 atom showed H-π interaction in binding with the TRP106 residue at a distance of 4.31 Å. The 6 ring of compound **2** demonstrated π-H interaction with LEU162 residue at a distance of 4.13 Å (Supplementary Table S14). 

Compound **3** resided in the active site of PL^Pro^; thereby, it mainly interacted with different residues through H–donor and developed H-π and π-H bonds (Figure 5C and Supplementary Figure S15A,B). Acting as H-donors, the two atoms O16 (3.03 Å) and O17 (3.66 Å) of compound **3** interacted with one of the catalytic triad (CYS111). Also, C27 (4.43 Å), O60 (4.61 Å), and C62 (4.35 Å) atoms of compound **3** bind to another residue (HIS272) of the catalytic triad through H-π interactions. Other atoms of compound **3,** like O10 (2.81 Å), O10 (3.11 Å), C34 (3.82 Å), and O52 (2.94 Å), acted as H-donors to interact with GLN269, TYR268, and CYS270 residues of PL^Pro^. The 6-ring of compound **3** showed π-H interaction with the LEU162 residue at a distance of 4.09 Å (Supplementary Table S15). 

Compound **4** showed its occupancy in the active site of PL^Pro^, where it interacted with the catalytic triad via H–donor and H-π interactions (Figure 5D and Supplementary Figure S16A,B). Compound **4** atoms C75 (3.86 Å), C17 (4.4 Å), and C22 (4.11 Å) exhibited their binding with one of the catalytic triad (HIS272) via H–donor and H-π interaction. Moreover, by means of H–donor, C65 showed binding with CYS270 at a distance of 3.83 Å (Supplementary Table S16).

Compound **5** docks in the active site of PL^Pro^, and it binds to the catalytic triad via H–donor behavior; also binds with other residues (Figure 5E and Supplementary Figure S17A,B). At a distance of 4.4 Å, by means of H-donor, the C75 atom of compound **5** binds to ASP286, a residue of the catalytic triad in the active site. In addition, the C10 atom of compound **5** also binds to another residue (CYS270) at a distance of 3.8 Å. Also, by the H-π interaction, C52 atoms showed binding with the TRP106 residue of PL^Pro^ (Supplementary Table S17).

Compound **6** stayed in the active site of PL^Pro^; there, it binds to the catalytic triad as well as other amino acids by means of H–donor, H-π, and π-H interactions (Figure 5F and Supplementary Figure S18A,B). HIS272, one of the amino acids of the catalytic triad, showed binding with C5 (4.08 Å) and the 6-ring (3.95 Å) of compound **6** by means of H-π and π-H interactions. Moreover, O19 and C34 atoms of compound **6** showed binding with CYS270 residue at a distance of 2.85 and 4.12 Å (Supplementary Table S18). 

**Figure 6:**
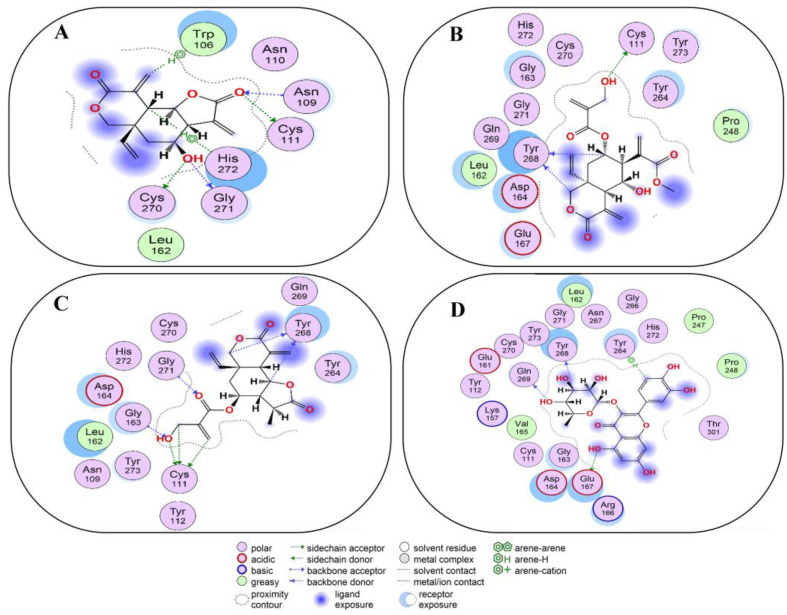
Two-dimensional view of PL^Pro^ showing non-covalent binding with compound **7** (**A**), compound **8** (**B**), compound **9** (**C**), and compound **10** (**D**). The surface representation and magnified view of compounds **1**–**6** in the active site of PL^Pro^ is shown in Supplementary Figures S19–S22. Images are generated by using MOE software.

Compound **7** docked into the active site of PL^Pro^, where it showed H–donor, H–acceptor, and H-π interactions with the catalytic triad, as well as interacted with other amino acid residues to establish bonds with them (Figure 6A and Supplementary Figure S19A,B). By means of H–donor and H-π interactions, the O23 and C17 atoms of compound **7** developed bonds with CYS111 and HIS272, two of the catalytic triad, at a distance of 3.26 and 4.21 Å, respectively. In addition, O23 atoms also established a bond with ASN109 at a distance of 3.46 Å. Likewise, O32 and C14 showed bond formation with CYS270, GLY271, and TRP106 residues at a distance of 3.3, 2.99, and 3.57 Å (Supplementary Table S19). 

Compound **8** also occupied the active site of PL^Pro^ and showed binding with the catalytic triad and other residues (Figure 6B and Supplementary Figure S20A,B). By means of H-donor and at a distance of 3.7 Å, the O48 atom of compound **8** showed binding with CYS111 of the catalytic triad. While C7 and C36 atoms showed binding with TRY268 residue by H–donor activity at a distance of 3.43 and 3.64 Å (Supplementary Table S20).

Compound **9** showed its occupancy at the active site of PL^Pro^. Compound **9** interacted with the catalytic triad and other important amino acids by means of H–donor and H–acceptor activities (Figure 6C and Supplementary Figure S21A,B). Regarding the catalytic triad, O7, C25, and C26 atoms of compound **9** exhibited interactions with the CYS111 residue of the catalytic triad at a distance of 3.75, 3.77, and 3.57 Å. In addition, compound **9** atoms like C11 (3.62 Å), C15 (3.34 Å), O6 (2.96 Å), and O7 (3.05 Å) interacted with TYR268, GLY271, and GLY163 amino acid residues of PL^pro^ (Supplementary Table S21).

Compound **10** exhibited its occupancy at the active site of PL^Pro^, where it preferentially interacted with amino acid residues as H–donor and H-π activities (Figure 6D and Supplementary Figure S22A,B). At a distance of 3.25, 3.34, and 2.9 Å, the O16, C45, and O51 atoms of compound **10** exhibited interactions with GLU167, TYR268, and GLN269 residues, which chiefly acted as H-donors. Only C31 of compound **10** interacted via H-π with TYR264 at a distance of 4.07 Å (Supplementary Table S22).

### 2.6. Covalent Docking of Compounds **1**–**10** 3CL^Pro^

The covalent binding occurred through the nucleophilic attack by 3CL^Pro^ and PL^Pro^ catalytic cysteine (CYS145/111) residues on the electrophilically potent sites of the compounds **7**, **8**, and **9**. To determine the covalent warheads we have implemented the density function approach to calculate the local descriptors for compounds **7, 8,** and **9** [40]. With the use of Gaussian (version 09W0) and Multiwfn (version 3.8) software, the local descriptors were calculated to determine the highly electrophilically potent sites (as covalent warheads) in compounds **7** (C13; electrophilicity index 3.217 eV), compound **8** (C19; electrophilicity index 2.535 eV), and compound **9** (C19; electrophilicity index 3.282 eV). Specifically, the CYS145/111 amino acids in the active sites of 3CL^Pro^ and PL^Pro^ targets the Michael acceptor double bonds (electrophilically potent sites) present in compound **7** (at C11 and C13), compounds **8** and **9** (C17 and C19), respectively (Supplementary Figure S23A). 

The atomistic and electronic level of the proposed covalent docking begins with the addition of ligands (**7**–**9**) to 3CL^Pro^ and PL^Pro^, considering protonated and deprotonated CYS145/111. This led to understand the equilibrium between CYS145/111-SH···HIS41/272 and CYS145/111-S−···+H-HIS41/272 protein patterns. Such ligand binding and eventual CYS145/111 deprotonation comprise a cycle, leading to covalent inhibition only once [Cys145/111-LIG]− is formed. This event is followed by proton transfer from the previously protonated HIS, ultimately restoring electron neutrality (Supplementary Figure S23B) [41]. 

**Figure 7:**
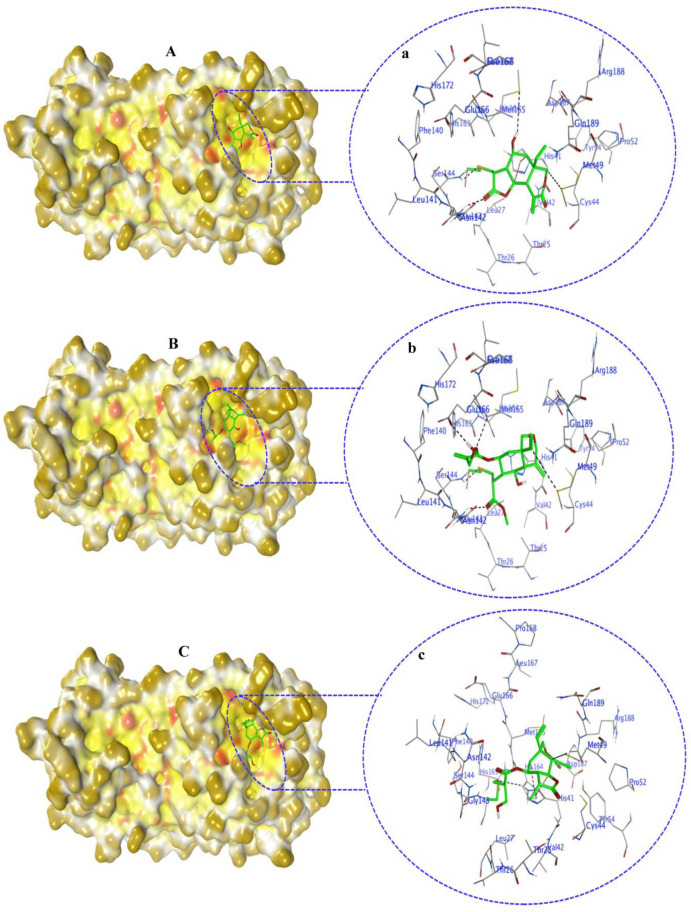
Compounds **7**, **8**, and **9** double-bond moieties and sulfur atoms of the CYS145 residue at the active site of 3CL^Pro^ exhibit covalent bond formation. Surface representation of 3CL^Pro^ docked with compound **7** (**A**), compound **8** (**B**), and compound **9** (**C**). The solvent-exposed region of 3CL^Pro^ is dark yellow, hydrophobic regions are yellow, and polar regions are red. Compounds **7**–**9** are in green. Magnified view of the 3CL^Pro^ active site occupied by compounds **7** (**a**), **8** (**b**), and **9** (**c**), showing their interactions with other amino acid residues in the active site. Bond colors in the magnified view of stick models are as follows: H-bond (black color), H-π bond (dark red), Van der Waals clashes (dark blue), atoms (element color), and residues are labeled as blue texts. Images are generated by using MOE software.

Covalent docking of 3CL^Pro^ with compounds **1**–**10** formed covalent bonds with only three compounds: vernolepin (compound **7**), vernodalol (compound **8**), and 11β,13-dihydrovernodalin (compound **9**). In compound **7**, the exocyclic double bond at positions 11 and 13 of the lactone ring acted as a warhead to develop a covalent bond with the sulfur atom (SG) of the CYS145 residue (Supplementary Figure S23). In addition to the covalent bonding, other important interactions also took place between compound **7** and 3CL^Pro^, such as the C17 atom of compound **7** developing a hydrogen bond with MET49 by means of a hydrogen donor at a distance of 3.68 Å. The sulfur (S8) and O33 atoms in compound **7** interacted with the nitrogen (N) atoms of CYS145 and GLY143 as a hydrogen acceptor at distances of 2.8 and 3.0 Å. On the other hand, C13 of the ligand developed a hydrogen bond with the 5-ring of HIS41 at a distance of 3.65 Å by means of H-π interaction (Figure 7(Aa)). The overall docking score for interaction between compound **7** and 3CL^Pro^ was −5.3 kcal/mol (Supplementary Table S23 and Figure S24).

The double bond at positions 17 and 19 in the side chain of compound **8** acted as a warhead, which covalently binds to the thiol group of the CYS145 residue in the active site of 3CL^Pro^ (Supplementary Figure S23). Moreover, compound **8** also exhibited interactions with other amino acid residues by H–donor and H–acceptor properties (Figure 7(Bb)). Compound **8** oxygen (O42) (3.21 Å, −0.6 kcal/mol) and sulfur (S8) (2.78 Å, −0.5 kcal/mol) atoms formed a hydrogen bond with CYS145 residue. Also, the oxygen (O42) (3.03 Å, −0.6 kcal/mol) atom of compound **8** formed a hydrogen bond with the nitrogen (N) atom of the GLU166 residue (Supplementary Table S24 and Figure S25). 

Moreover, in compound **9**, the double bond moiety at positions 17 and 19 of the side chain acted as a warhead that formed a covalent bond with the sulfur atom of the CYS145 residue (Supplementary Figure S23). Compound **9** also interacted with other residues of 3CL^Pro^ as a hydrogen bond donor, acceptor, and H-π (Figure 7(Cc)). The compound **9** C33 atom (3.8 Å, −0.4 kcal/mol) developed a hydrogen bond by donating hydrogen to the sulfur (SD) atom of the MET49 residue in the receptor. A hydrogen bond also developed between the S8 atom (3.79 Å, −0.8 kcal/mol) and the nitrogen atom (NE) of HIS41 residue. C19 (4.01 Å, −0.5 kcal/mol) and C35 (4.61 Å, −0.7 kcal/mol) atoms formed hydrogen bonds with the 5-ring of HIS41 by means of H-π interactions (Supplementary Table S25 and Figure S26).

### 2.7. Covalent Docking of Compounds **1**–**10** with PL^Pro^

**Figure 8:**
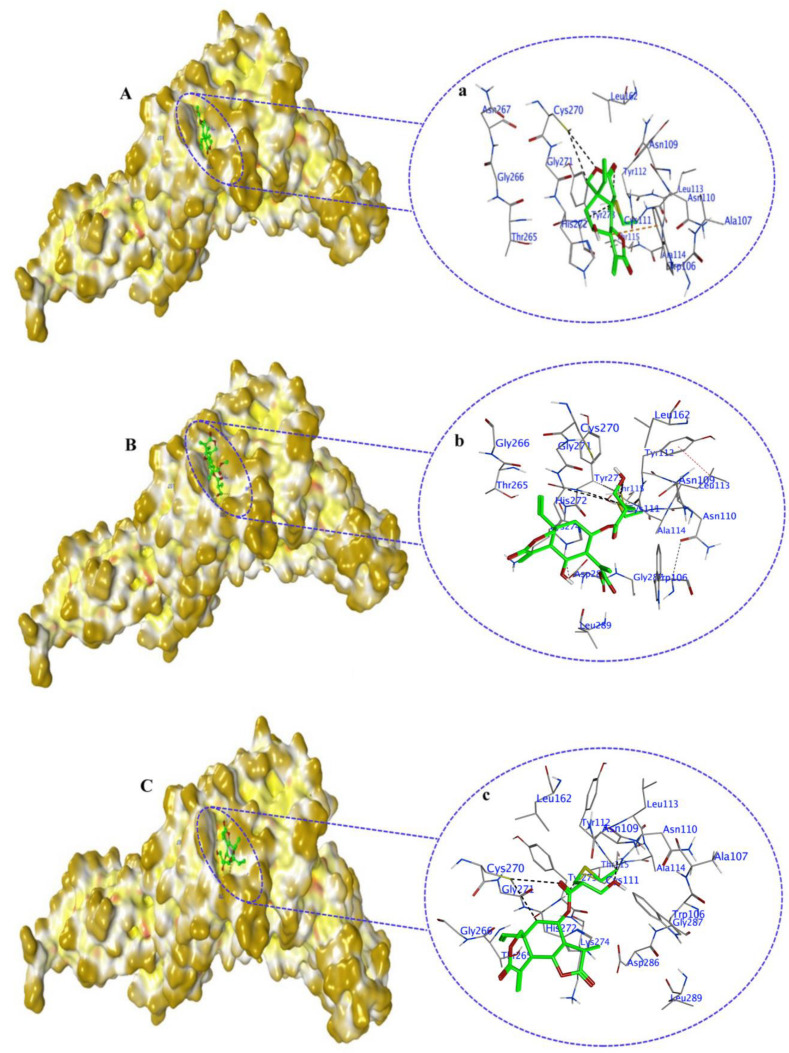
Compounds **7**, **8**, and **9** double-bond moieties and sulfur atoms of the CYS111 residue at the active site of PL^Pro^ exhibit covalent bond formation. Surface representation of PL^Pro^ docked with compound **7** (**A**), compound **8** (**B**) and compound **9** (**C**). The solvent-exposed region of PL^Pro^ is dark yellow, hydrophobic regions are yellow, and polar regions are red. Compounds **7**–**9** are in green. Magnified view of the PL^Pro^ active site occupied by compounds **7** (**a**), **8** (**b**), and **9** (**c**), showing their interactions with other amino acid residues in the active site. Bond colors in the magnified view of stick models are as follows: H-bond (black color), H-π bond (dark red), Van der Waals clashes (dark blue), atoms (element color), and residues are labeled as blue texts. Images are generated by using MOE software.

Covalent docking of PL^Pro^ with compounds **1**–**10** resulted in covalent bond formation only with compounds **7**, **8**, and **9**. Also, some other interactions occurred between these three compounds and PL^Pro^. In compound **7**, the double bond at positions 11 and 13 of the lactone ring developed a covalent bond with a sulfur atom of CYS111 in the active site of PL^Pro^ (Figure 8(Aa)). In addition, compound **7**, as a ligand, also developed another type of interactions with amino acid residues in PL^Pro^. The C15 atom of compound **7** acted as a hydrogen donor to the sulfur of CYS270 for hydrogen bond formation at a distance of 3.75 Å with a binding energy of −0.5 kcal/mol. Also, sulfur (S8) atom of compound **7** developed a hydrogen bond by accepting hydrogen from nitrogen (N) of the TRY273 residue at a distance of 4.07 Å, having a binding energy of −2 kcal/mol. Moreover, the H-π interaction between the C37 atom of compound **7** and the 6-ring of TRP106 showed a hydrogen bond between them. Overall, the docking score for this interaction was −4.4 kcal/mol (Supplementary Table S26 and Figure S27).

As a ligand, the compound **8** double bond at positions 17 and 19 in the side chain also developed a covalent bond with the sulfur atom of CYS111. Moreover, compound **8** also developed hydrogen bonds with other amino acid residues in the target protein (Figure 8(Bb)). As a hydrogen donor, the oxygen (O57) atom of compound **8** established hydrogen bonds with OD1 of ASN109 and sulfur (SG) of CYS270 at a distance of 2.75 and 4.02 Å, and the interaction energy was found to be 2.8 and 4.0 kcal/mol. However, the sulfur (S8) of compound **8** accepted hydrogen atoms from nitrogen (N) of CYS111 and TYR273 amino acid residues to develop hydrogen bonds at a distance of 2.87 and 4.49 Å. Hydrogen bonds also developed through H-π interactions between the C25 atom and 5-ring of TRP106, C31 atom and 6-ring of TRP106. The overall docking score for the interaction between compound **8** and PL^Pro^ was −4.9 kcal/mol (Supplementary Table S27 and Figure S28). 

Furthermore, a covalent bond also developed between the sulfur atom of CYS111 and the double-bond moiety of compound **9** at positions 17 and 19 in the side chain (Figure 8(Cc)). Other important interactions also developed between compound **9** and PL^Pro^. O14 and C21 atoms of compound **9** donated hydrogen to the sulfur atom of CYS270 in PL^Pro^ to form hydrogen bonds at a distance of 3.77 and 3.87 Å, with binding energies of −0.9 and −0.5 kcal/mol, respectively. Additionally, sulfur (S8) and oxygen (O15) atoms of compound **9** accepted hydrogen from nitrogen (N) of TRY273 and CYC111 amino acid residues to form hydrogen bonds at a distance of 4.11 (−1 kcal/mol) and 3.04 Å (−1 kcal/mol). The overall docking score for the interaction between compound **9** and PL^Pro^ was −4.7 kcal/mol (Supplementary Table S28 and Figure S29).

### 2.8. MD Simulation Analysis

#### 2.8.1. Root-Mean-Square-Deviation (RMSD) of Compounds **7**, **8**, and **9** with 3CL^Pro^ and PL^Pro^

**Figure 9:**
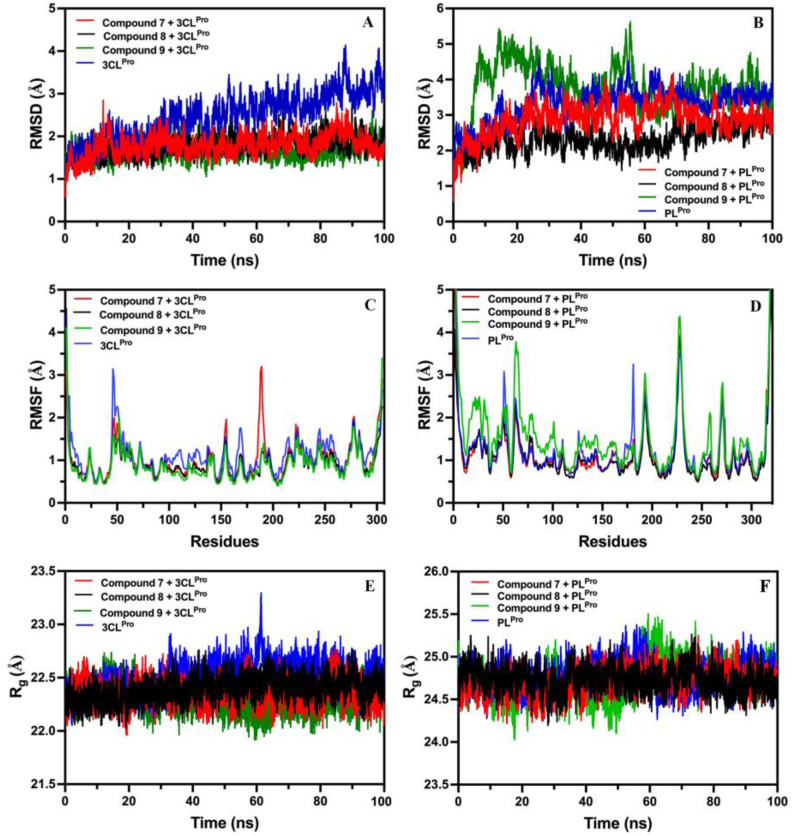
RMSD of unbound 3CL^Pro^ (**A**), PL^Pro^ (**B**), and during their complexation with compounds **7**, **8**, and **9** as a function of time. RMSF of 3CL^Pro^ (**C**) and PL^Pro^ (**D**) shows the flexibility and stability of amino acid residues in the absence and presence of ligands (compounds **7**, **8**, and **9**). Rg of 3CL^Pro^ (**E**) and PL^Pro^ (**F**) exhibiting the compactness of amino acid residues in the absence and presence of ligands (compounds **7**, **8**, and **9**). The data for RMSD, RMSF and Rg generated from MD simulations were replotted using GraphPad Prism 9.

The stability and conformational dynamics of 3CL^Pro^ with compounds **7**, **8**, and **9** were further analyzed using MD simulations by measuring the RMSD, a parameter explaining the conformational variations in and stability of biomolecules. The RMSD of the backbone 3CL^Pro^ was compared with the RMSD of the 3CL^Pro^ complexed with compounds **7**, **8**, and **9** to understand the complexes’ stability. RMSD representing the fluctuations in the average position of ligands (compounds **7**, **8**, and **9**) and 3CL^Pro^ are shown in Figure 9A. The RMSD values exhibited the average deviation of compounds **7**, **8**, and **9** positions from their initial reference structure during 100 ns. RMSD data exhibited that compounds **7**, **8**, and **9** formed relatively stable interactions with 3CL^Pro^. Compound **7** displays an average RMSD value of 1.778 ± 0.253, while compound **8** showed a slightly lower average deviation in the RMSD value (i.e., 1.763 ± 0.226). Compound **9** showed an average deviation of 1.658 ± 0.211 Å. Overall, the RMSD of compounds **7**, **8**, and **9** were near to each other, and comparable to the RMSD value of unbound 3CL^Pro^ (2.466 ± 0.539 Å).

The RMSD of PL^Pro^ was related to the RMSD of PL^Pro^ bound to compounds **7**, **8**, and **9**. Fluctuations in the average position of PL^Pro^ and ligands (compounds **7**, **8**, and **9**) bound to PL^Pro^ are shown in Figure 9B. RMSD values exhibited an average deviation of compounds **7**, **8**, and **9**’s positions from their initial reference structure during 100 ns. Compounds **7**, **8**, and **9** formed relatively stable interactions with PL^Pro^. Compound **9** showed an average deviation of 3.777 ± 0.690 Å, while compound **8** showed a slightly lower average deviation in the RMSD value (i.e., 2.316 ± 0.356). Compound **7** displayed an average RMSD value of 2.877 ± 0.438. Overall, the RMSDs of compounds **7**, **8**, and **9** were close to each other and comparable to the RMSD value of unbound PL^Pro^ (3.313 ± 0.479 Å). The standard deviation (SD) of RMSD values was found to be less than the PL^Pro^, indicating decreased fluctuation and stable complex formation as compared to protein alone.

#### 2.8.2. Root-Mean-Square-Fluctuation (RMSF) of Compounds **7**, **8**, and **9** with 3CL^Pro^ and PL^Pro^

3CL^Pro^ and PL^Pro^ target proteins were further analyzed by RMSF measurements to determine the flexibility and stability of their amino acid residues in the active site when bound to ligands (compounds **7**, **8**, and **9**), and fell within the acceptable range. RMSF analysis of unbound 3CL^Pro^ exhibited small spikes of fluctuation, except for a few regions encompassing residues like 1–10, 45–62, 70–74, 93–120, 131–147, 156–171, 188–200, 210–262, 273–286, and 290–300. These regions are associated with the loop region in 3CL^Pro^, which is more dynamic and less constrained relative to the rest of the protein structure. However, compounds **7**, **8**, and **9,** binding with 3CL^Pro^ resulted in greater stabilization, as well as decreasing the amino acid fluctuations during 100 ns MD simulations (Figure 9C). The RMSF plot of unbound PL^Pro^ provides insights into the fluctuations and flexibility of residues during the 100 ns trajectory, especially residues 1–5, 49–53, 61–65, 179–182, 191–196, 224–232, 268–272, and 314–321. These fluctuations were due to their location inside the loop regions, which have greater flexibility. In contrast, PL^Pro^, when complexed with compounds **7** and **8**, demonstrated lower fluctuations that provided stability to the interacting residues in this region, while compound **9** showed high fluctuation (Figure 9D).

#### 2.8.3. Radius of Gyration (Rg) of Compounds **7**, **8**, and **9** with 3CL^Pro^ and PL^Pro^

Rg analysis was evaluated to determine the compactness of amino acids and conformational changes in 3CL^Pro^ and PL^Pro^. To achieve this, Rg of 3CL^Pro^ and PL^Pro^ were analyzed in the presence and absence of compounds **7**, **8**, and **9**. 3CL^Pro^, in the absence of ligands, exhibited an Rg value of 22.508 Å. However, when 3CL^Pro^ bound to compounds **7**, **8**, and **9,** the Rg value decreased to 22.346, 22.393, and 22.298 Å, respectively (Figure 9E). Also, PL^Pro^ in the unbound state showed an Rg of 24.776 Å that was reduced to 24.705, 24.731, and 24.732 Å after the binding of compounds **7**, **8**, and **9** (Figure 9F).

## 3. Discussion

3CL^Pro^ is one of the most widely studied and prominent targets for the development of novel drugs against SARS-CoV-2. 3CL^Pro^ is highly conserved and shows 100% sequence similarity in the active site with its counterpart (SARS-CoV) [42,43]. As a homodimer protease, each monomer of 3CL^Pro^ comprises 306 amino acid residues and has three domains. The active site of 3CL^Pro^ contains a catalytic dyad (HIS41 and CYS145). Other amino acid residues in active site responsible for substrate binding are SER144, HIS163, GLN192, GLU143, HIS41, LEU167, ALA191, GLU166, MET165, MET49, THR190, ASP187, HIS164, GLN189, and ARG188. 3CL^Pro^ mediates vital processes required for the survival of the virus, including genome replication and transcription. There is no analogous protease in humans; hence, 3CL^Pro^ has been identified as a promising target for drug development against SARS-CoV-2 [44]. 

PL^Pro^, a large multidomain protein, is a crucial element of the SARS-CoV-2 replicase transcriptase complex. PL^Pro^ mediates the vital activity of proteolytic processing and viral maturation [11,45,46]. PL^Pro^ is highly conserved, contains a large amount of cysteine (3.5%), and is composed of 316 amino acid residues [47]. The active site of PL^Pro^ contains a catalytic triad (CYS111, HIS272, and ASP286) [48]. There are two zinc (Zn) ions that bind to PL^Pro^, which is mainly coordinated by four cysteine residues (CYS189, CYS192, CYS224, and CYS226). The binding of Zn ions is crucial for the structural stability as well as the protease activity of PL^Pro^ [11]. Moreover, GLY271, GLN269, LEU162, TYR273, PRO247, PRO248, TYR268, TYR264, ASN267, CYS270, THR301, and MET208 are also responsible for substrate binding in PL^Pro^ [49].

One of the common approaches in medicinal drug discovery and development is to focus on a single target, which mainly relies on finding an inhibitor that exhibits a high binding affinity for the target. Nonetheless, such attempts do not always succeed. Rather, a multi-targeted approach is much more preferred, which chiefly relies on the partial inhibition of several targets by one compound [11]. In this condition, a high binding affinity is not a thumb rule for the inhibitor. In fact, the partial inhibition of multiple targets has demonstrated more efficacy than the complete inhibition of a single target [11,50]. Hence, we chose those ten phytochemicals (compounds **1**–**10**), which we isolated, purified, characterized in our lab, and evaluated for their drug-like efficacy in different test systems [33,34,35]. Relying on the properties of compounds **1**–**10**, we used computer-aided in silico analysis of the inhibitory effects of these compounds on dual targets (3CL^Pro^ and PL^Pro^) of SARS-CoV-2. To that end, we first analyzed the pharmacokinetics of compounds **1**–**10**. These compounds exhibited strong pharmacokinetic characteristics and maintained the Lipinski rules of five (RO5), indicating their suitability as future drug molecules [51,52]. The toxicological properties of compounds **1**–**10** with good ADME characters were used to evaluate their adverse effects on humans [52]. No toxicity was found for compounds **1**–**10**, and these compounds showed good solubility, and intestinal permeability, which provided them easy penetration into the intestinal wall or blood stream for their further action [53]. 

We then validated the docking protocol to ensure that the data obtained from the docking protocol with ligands X77 and 3CL^Pro^ and VIR250 and PL^Pro^ demonstrated stability, specificity, and conformity. Overall, the interaction between the mentioned ligands and their receptors unequivocally demonstrated the utility and reliability of the docking protocol used in our study. Subsequently, we were encouraged to evaluate the type of interaction between compounds **1**–**10** with 3CL^Pro^ and PL^Pro^. To do so, we performed two types of docking analysis. Firstly, a non-covalent (reversible) docking protocol was implemented to identify inhibitors among compounds **1**–**10** that interact in the active sites of 3CL^Pro^ and PL^Pro^. Secondly, a covalent (irreversible) docking protocol was implemented to find inhibitors among compounds **1**–**10** that develop a covalent bond within the active sites of 3CL^Pro^ and PL^Pro^. 

We found that the non-covalent docking data of compounds **1**–**10** specify some pertinent modes of interactions like H–donor, H–acceptor, H-π, and π-H, as well as π-π stacking. Compounds **1**–**10** interacted with catalytic dyads (CYS145 and HIS41) in the active site of 3CL^Pro^. In spite of such selective binding, compounds **1**–**10** also possess the potential to bind with other important residues in the active site of 3CL^Pro^. These interactions contributed substantially to the binding potential of compounds **1**–**10** with 3CL^Pro^. The non-covalent interaction of compounds **1**–**10** with 3CL^Pro^ is in agreement with some recent studies in which phytochemicals and herbal medicines have also shown their preferential binding with catalytic dyads that led to the inhibition of 3CL^Pro^ activity in SARS-CoV-2 [29,54,55]. In addition, we found that compounds **2**, **3**, and **10** showed high negative docking scores (−7.7, −8.0, and −8.2 kcal/mol), which are comparable to the Remdesivir-3CL^Pro^ docking score (−8.2 kcal/mol) [56]. The docking score determines the most favorable conformation of a ligand to establish binding in the active site of a receptor. A preferred binding conformation also influences the overall binding affinity. Hence, a lower docking score results in a more favorable conformation between the ligand and receptor [57]. 

Non-covalent docking data of compounds **1**–**10** demonstrated their ability to occupy the active site of PL^Pro^. In particular, compounds **1**, **2**, **3**, **4**, **5**, **6**, and **7** interacted with the catalytic triad (CYS111, HIS272, and ASP286), which are crucial amino acids in the active site of PL^Pro^ [11,48]. In addition, compounds **1**–**7** and **10** atoms also exhibited their interactions with other amino acids (THR301, CYS270, LEU162, GLN269, and TRY264) of PL^Pro^, which are involved in substrate binding [49,58]. SARS-CoV-2 PL^Pro^ comprises four main sub-domains, including the ubiquitin-like domain (N-terminal), thumb domain (α-helical), finger domain (β-stranded), and palm domain. The ubiquitin-specific protease deubiquitinating enzyme (DUB) demonstrated low homology with SARS-CoV-2 PL^Pro^ [49]. There are six α-helices and a small β-hairpin in the thumb domain. On the other hand, six β-strands and two α-helices make the finger subdomain. In the palm subdomain, there are six β-strands. Intriguingly, the DUB and proteolytic sites of SARS-CoV-2 PL^Pro^ are self-governing and suggest two possible activities of PL^Pro^. The catalytic triad (CYS111, HIS272, and ASP286) is typically found at the interface of the palm and thumb subdomains. In addition to the catalytic triad, there are three additional residues that play a crucial role in SARS-CoV-2 PL^Pro^ enzymatic activity. The β-turn/loop (GLU266-GLY271) close to the binding of the inhibitor or substrate is located along the side of the active site. TYR268, being a part of GLU266-GLY271, mediates a vital process of proteolytic activity, and GLU167 also plays an important role in ubiquitin core recognition. Mutation in TYR268 has displayed obstructions in the proteolytic activity of SARS-CoV-2 PL^Pro^, while GLU167 mutation leads to the abrogation of DUB activity [49,59]. Hence, any molecule that forms a hydrogen bond either with GLU167 or TYR268 has the capability to interfere with the proteolytic activity and may abrogate SARS-CoV-2 PL^Pro^ DUB activity [59]. Within the cohort of tested compounds **1**–**10**, their capability of interaction and establishment of H-bonds either with the catalytic triad or other amino acids indicated that they may show inhibitory activity against SARS-CoV-2 PL^Pro^.

The promising data of the non-covalent (reversible) interaction of compounds **1**–**10** with 3CL^Pro^ and PL^Pro^ motivated us to further analyze covalent binding (irreversible) patterns with the indicated targets. In this regard, CYS145 has been identified previously as a crucial residue within the active site of 3CL^Pro^, signifying it as a potential target for covalent inhibitors [60,61]. Covalent docking has been categorized into two stages: firstly, to identify a candidate ligand with a proper pose as its reactive group near CYS145, and secondly, a simulated chemical reaction between the reactive groups that leads to the development of a stable covalent bond [60,61]. Within these criteria, those ligand poses that fall within the distance cut-offs (reacting pairs of atoms within 5) were retained, and a covalent bond (S–C) was established by virtue of the reaction taking place. To determine the prospective covalent inhibitor from compounds **1**–**10**, all parameters from the MOE program, like Michael’s addition reaction, nucleophilic addition to double and triple bonds, nucleophilic substitution, and aryl- and nitrile-activated conjugate addition to alkynes, were implemented. Following these measures, compounds **1**–**10** were subjected to covalent docking with 3CL^Pro^, which revealed that only three compounds (**7**, **8**, and **9**) carry the covalent warheads that resulted in covalent bond formation through Michael acceptors (Supplementary Figure S23). The covalent warhead in compound **7** is the exocyclic double bond at positions 11 and 13 of the lactone ring. In compound **8**, the double bond at positions 17 and 19 of the side chain is the covalent warhead. In compound **9**, the covalent warhead is the double bond moiety (positions 17 and 19) of the side chain. In addition, these compounds also established H-bond formation with certain residues in the active site. Compounds **7**, **8**, and **9** formed covalent bonds with the thiol group of the CYS145 residue in the active site of 3CL^Pro^. In cysteine (CYS), the large atomic radius of sulfur (S) and low dissociation energy of the S-H bond render its thiol group to possess the capability of nucleophilic and redox active functions. Hence, relative to other amino acids, CYS is the most common covalent amino acid residue in the development of covalent drug inhibitors [62,63]. Especially CYS reacts with a range of bioactive products derived from plants that contain natural warheads, including Michael receptors and compounds containing electrophilic groups in their structure [64]. The molecular docking data suggested two possible variants for binding of compounds **7**, **8**, and **9**, stable or irreversible covalent binding and reversible inhibition by non-covalent binding with 3CL^Pro^, indicating that these compounds may disrupt the function of the target protein and reduce viral replication [65]. In the case of PL^Pro^, a covalent bond was formed by compounds **7**, **8**, and **9** with the thiol group of CYS111 via Michael reaction acceptors. Moreover, these compounds also developed reversible (non-covalent) interactions with other amino acid residues within the active site of PL^Pro^. The dual mode of interaction of compounds **7**, **8**, and **9** unequivocally indicates their potential to inhibit the activity of PL^Pro^. Similar to our finding, peptide inhibitors (VIR250 and VIR251), as well as small molecules (GRL0617 and rac5c), exhibited irreversible bond formation with CYS111 in the active site to inhibit PL^Pro^ activity [66,67,68]. Overall, viewing the reversible and irreversible binding of compounds **1**–**10** with the two targets (3CL^Pro^ and PL^Pro^), it can be inferred that the structure–activity relationship (SAR) has played a crucial role that may enable them to act as strong inhibitors and may affect viral replication (Supplementary Tables S29 and S30). Similar to our findings, earlier studies have also reported the crucial role of SAR and the inhibitory effects of drugs on 3CL^Pro^ and PL^Pro^ [69,70,71,72]. 

We further assessed the impact of compounds **7**, **8**, and **9** on the structural and conformational changes in 3CL^Pro^ and PL^Pro^, which may offer the potential inhibitory effects of selected compounds. Hence, we performed atomic-scale MD simulations to unravel the stability of compounds **7**, **8**, and **9** with 3CL^Pro^ and PL^Pro^. In this regard, RMSD analysis was performed to measure changes in the simulated structures of target proteases in the absence and presence of ligands (compounds **7**, **8**, and **9**) [73]. We have found that three compounds (**7**–**9**) exhibited little change when fit to 3CL^Pro^. Two compounds (**7** and **8**) showed little change (i.e., more rigid) with PL^Pro^, while compound **9** showed an increase in fluctuation when fit to PL^Pro^, indicating less stability. Overall, the RMSD of compounds was somewhat lower or near the RMSD of the target proteins. Such characters unequivocally indicate the reality that tested compounds, as ligands, were stably attached to the active sites. Similar findings on phytochemicals showed little variation in RMSD, affirming their stability in the active site of 3CL^Pro^ and PL^Pro^ [11,74]. 

We then quantitated the RMSF to understand the flexibility per residue in the active sites of 3CL^Pro^ and PL^Pro^ in unbound and bound conformations with ligands (compounds **7**, **8**, and **9**) [75]. In the absence of ligands, amino acid residues in the active sites of 3CL^Pro^ and PL^Pro^ showed higher flexibility, primarily due to the loop regions and terminal residues, which are dynamic and less controlled [11,76,77]. We found that the per-residue RMSF of ligand–target protein complexes was either reduced or showed nearby fluctuation levels, as observed in the unbound target, and it is in accordance with the fact that smaller-ligand RMSFs are deemed better [78]. Consequently, our RMSF data affirmed that compounds **7**, **8**, and **9** retain the inherent potential to maintain stable interaction throughout the MD simulation time, which is also crucial for the assessment of inhibitors against SARS-CoV-2. Similar to our findings, in silico analysis of phytochemicals showed their effectiveness in stabilizing the fluctuations per residue in the above targets of SARS-CoV-2 [11,79]. Lastly, we focused our analysis on measuring the radius of gyration (Rg) of 3CL^Pro^ and PL^Pro^ during unbound and bound states to the ligands (compounds **7**, **8**, and **9**). We found that Rg analysis mirrored the effects of the RMSD and RMSF by lowering the Rg values of amino acid residues in the active sites of their respective targets. Moreover, it is also envisaged that the compactness of 3CL^Pro^ and PL^Pro^ did not change, and ligands did not dissociate from the targets during the MD simulation period; in fact, the target was more stabilized upon their complexation with compounds **7**, **8**, and **9** [11,79]. 

## 4. Materials and Methods

### 4.1. Phytochemicals from Medicinal Plants

Ten compounds (**1**–**10**) isolated previously in our lab from Arabian Peninsula medicinal plants were used for the current in silico study. The 2D structure of compounds (**1**–**10**) was drawn with MarvinSketch [80]. The 3D structure of compounds (**1**–**10**) was prepared by the Molecular Operating Environment (MOE)-builder tool, a part of MOE suit (MOE version 2015.10 software) (Chemical Computing Group Inc., Montreal, Canada). Details of each compound are listed in the Section 2 (Table 1).

### 4.2. Drug-Likeness and ADMET Properties

The drug-likeness properties of compounds **1**–**10** were analyzed by BIOVIA Discovery Studio 4.5 (D.S.4.5) (San Diego, California, United States). Compounds **1**–**10** were first quantified for Property Calculation by selecting the Calculate Molecular Opting Properties in which different parameters present in 2D were selected and the analysis was performed. The drug-likeness of compounds **1**–**10** was evaluated based on Veber and Lipinski’s rule of five (RO5). In this perspective, we evaluated compounds **1**–**10** for possessing a molecular weight < 500 Da, having an ALog P < 5 (lipophilicity), containing a number of hydrogen-bond acceptors (nHBA) <10, and a number of hydrogen-bond donors (nHBD) <5 [51,52,81]. Subsequently, 2D data for compounds **1**–**10** were imported into the BIOVIA Discovery Studio. Minimization of compounds **1**–**10** was performed in the In Situ Ligand Minimization module using the MMFF option in the Input Forcefield. The rest of the minimization parameters were kept at their default values. Post-minimization, compounds **1**–**10** were subjected to the Calculate Molecule Properties option. Afterwards, an ADMET descriptor was run to predict different parameters of compounds **1**–**10** as well as plots of AlogP (ADMET AlogP98) and 2D polar surface area (PSA_2D) were developed. The hepatotoxicity of compounds **1**–**10** was calculated using ProTox-II [82]. In addition, two descriptors, i.e., the topological surface area (TPSA), ranging between 20 and 140 Å^2^, and molar refractivity (MR), ranging between 40–130, of compounds **1**–**10** were calculated using the Swiss ADME server [83]. Compounds showing a violation of two (2) or more were considered less drug-like characters [52]. 

### 4.3. Ligand Preparation

The 2D structures of compounds **1**–**10** were drawn using MarvinSketch (Chemaxon, Budapest, Hungary). The ligand preparation of compounds **1**–**10** was developed using the Molecular Operating Environment (MOE)-builder tool. Additionally, MMFF994x was used to minimize the energy of compounds **1**–**10** up to the conjugate gradient root-mean-square (RMS) to be <0.05 kcal/mol Å^−1^. Moreover, the electrostatic potential (ESP) PM3 and AM1 methods were implicated to calculate the partial charges, as well as the atomic charges (partial) for ligands and atoms being calculated.

### 4.4. Target Protein Preparation

The crystal structures of SARS-CoV-2 3CL^Pro^ (PDB ID: 6W63) and PL^Pro^ (PDB ID: 6WUU) were downloaded from the Protein Data Bank [84]. The 3CL^Pro^ resolution was 2.10 Å, and X77 was a co-crystalized ligand in this protein. The PL^Pro^ resolution was 2.79 Å, and the VIR250 inhibitor was co-crystalized into it. Water was removed from the above protein targets, and the structure preparation module was used to correct the protein, which was simultaneously 3D protonated. Afterward, the active sites of the above target proteins were identified by the use of co-crystalized ligands. Before the application of the preliminary docking protocol, the above-mentioned co-crystalized ligands were re-docked using the docking protocol on the protein-binding site to validate the protocol. To accept the protocol of docking, the root-mean-square deviation (RMSD) value was observed for <3 for each of the co-crystalized ligands and redocked ligands using the MOE scientific vector language script [85].

### 4.5. Docking Experiments

#### 4.5.1. Validation of Docking

SARS-CoV-2 protease crystals of 3CL^Pro^ and PL^Pro^ provide the structural knowledge for the docking-based identification of promising phytochemical compounds in this study. By using MOE software (Chemical Computing Group Inc., Montreal, Canada), validation of docking was performed with 3CL^Pro^ and PL^Pro^, which were co-crystallized with ligands. Subsequently, the ligands were re-docked into the active site as per the protocol of docking to confirm their alignment with the protocol and active site confirmation [86]. Within the MOE software, ligand atoms, triangle matcher, London dG, and GBVI/WSA dG were selected for the fields of site, method (placement), and score, respectively. All parameters were kept at their default values. In each cycle of the triangle matcher, the best conformation of ligand showing a high score and suitable orientation was selected.

#### 4.5.2. Non-Covalent Docking

The non-covalent docking of compounds **1**–**10** with 3CL^Pro^ and PL^Pro^ was performed using the MOE software suite. Non-covalent docking was started with the preparation of 3CL^Pro^ and PL^Pro^. The target proteins were subjected to protonation using the protonate 3D method [87]. Subsequently, the target proteins were partially charged using an MOE-implemented AMBER10:EHT force field. After protonation and charge addition, 3CL^Pro^ and PL^Pro^ were run for non-covalent docking with compounds **1**–**10** implementing the identical protocol used for the validation of docking. Docking results were generated in a database file (.mdp) that was browsed in MOE software. The main bond for the analysis of interaction between compounds **1**–**10** and both targets (3CL^Pro^ and PL^Pro^) were H-bond. The length of H-bond was automatically measured by the MOE software between the heavy atoms of compounds **1**–**10** and the mentioned targets. Consequently, the distances exceeded the typical range for hydrogen bonds (2.8–3.0Å). Therefore, the reported distances in our analysis were greater than 3.5 Å, but they were consistent with the actual spatial arrangement of the molecules when considering the positions of the hydrogen atoms. For the π-H and H-π interactions, we considered both polar and non-polar hydrogens, specifically focusing on their unique contributions and energy profiles. Polar hydrogens were typically above the energy cutoff (−0.5 kcal/mol), while non-polar hydrogens were less than the energy threshold (−0.5 kcal/mol). 

#### 4.5.3. Covalent Docking

Covalent docking of compounds **1**–**10** was performed using the covalent docking module within the MOE software suite. The 3CL^Pro^ and PL^Pro^ were prepared, protonated, and partially charged using the MOE-implemented AMBER10:EHT force field, as performed within the non-covalent docking protocol. For the covalent docking protocol, a selection of reactive sites was performed, which showed cysteine 145 (CYS145) and cysteine 111 (CYS111) residues in 3CL^Pro^ and PL^Pro^ targets. The sulfur (S) atom of thiol groups (-SH) in CYS145 in 3CL^Pro^ and CYS111 in PL^Pro^ was selected as a reactive site in the covalent docking. 

The covalent docking was performed by implementing the Michael Addition Reaction between the thiol groups (-SH) in CYS145/111 and warheads of ligands (compounds **7**–**9**). The chiral centers resulting from the Michael addition reactions had their chirality attributed by the MOE software suite. The covalent warheads of ligands (compounds **7**–**9**) were determined by a density function approach by calculating the local descriptors using Gaussian and Multiwfn software, which calculated the highly electrophilically potent sites (as covalent warheads) in compounds **7** (C13; electrophilicity index 3.217 eV), **8** (C19; electrophilicity index 2.535 eV), and **9** (C19; electrophilicity index 3.282 eV), respectively [40]. The best conformation of ligands that showed their ability to form covalent bonds, along with high scores, were selected for further studies [88,89,90,91]. 

### 4.6. Molecular Dynamics (MD) Simulations

The best docking pose from covalent docking was selected for MD simulations. MD simulation was started by performing the energy minimization of three complexes with 3CL^Pro^ and PL^Pro^ using the MOE software suite. Minimization was carried out by employing the Assisted Model Building with Energy Refinement (AMBER 10) package as well as Merck molecular force field static (MMFF94s) [92]. The system topology was created by the CHARMM-GUI solution builder module. CHARMM36 [93] and CHARMM General Force Field (CGenFF) [94,95,96] were, respectively, used to describe the proteins (3CL^Pro^ and PL^Pro^) and the small ligands (**7**–**9**), in both their complexed and native target forms. For the forcefield parameters, the PDB files of 3CL^Pro^ and PL^Pro^ with compounds **7**, **8**, and **9** were uploaded in the solution builder option of CHARMM-GUI. Afterwards, in the model/chain selection option, all checkboxes were selected and moved to the next step of the PDB manipulating option, where the option of ‘add covalent bond’ emerged only when the ligand complexes were covalently bonded (Supplementary Figure S30). Subsequently, clicking the edit button leads to the appearance of a dialogue box titled ‘Chemical structure of virtual ligand’ in ChemAxon online software. Using this option, ligands (compounds **7**–**9**) were edited to add a covalent bond with the thiol (SH) of CYS 145/111 in 3CL^Pro^ and PL^Pro^ for parameterization (Supplementary Figure S31). The target amino acid residues (CYS 145/111) and the ligands (compounds **7**–**9**) were parameterized together as LIG(C13/C19/C19→SG)CYS in the option to add a covalent bond. The option of the next step was clicked to generate the PDB files that generated the topology and parameter files of the ligands (Supplementary Figure S32).

Further, the transferable intermolecular potential water molecules (TIP3P) model was implemented to solvate all systems [97]. An octahedral computational box, having a 12 Å distance between the edges, was employed for protein solvation [82]. The system was neutralized by the addition of sodium (Na) and chloride (Cl) ions, with a final concentration of 0.15 M. Equilibrations were performed in three stages (minimize, number of steps, and run). The indicated stages were accomplished using the following values: 10,000 (minimize), 1,000,000 (number of steps), and 125,000 (run). Equilibration simulation in the canonical ensemble (NVT) was performed for 125 ps. All systems were simulated at a temperature of 310 K, which was maintained with a constant time of 1 ps. The 100 ns of production simulations were performed using NPT ensemble (constant number of particles, pressure, and temperature). In this process, the pressure was 1atm, and the control of processing was run with a constant 5 ps. NAMD 3.0 was used for all MD simulations, and trajectories were visualized on VMD packages. The short simulation (100 ns) time was considered the time at which biological phenomena occur. Most importantly, equilibrium was reached in the RMSD at 100 ns in the production phase. Hence, we studied the simulation for 100 ns. It is important to mention that our 100 ns study is not an absolute result, and it is not certain that there will be any change or not by extending time. The root-mean-square deviation (RMSD), root-mean-square fluctuation (RMSF), and radius of gyration (Rg) were analyzed on VMD packages [98].

## 5. Conclusions

Herein, we evaluated the in silico inhibitory potential of compounds **1**–**10** against 3CL^Pro^ and PL^Pro^ targets in SARS-CoV-2. In order to do so, compounds **1**–**10** were first evaluated for their drug-likeness properties based on the criteria of Lipinski’s rule of five (RO5). Compounds exhibited strong pharmacokinetic characteristics and maintained RO5, indicating their suitability as future drug molecules. Furthermore, in silico prediction of compounds **1**–**10** for ADMET behavior were performed. ADMET data showed that all compounds were non-hepatotoxic and showed low to good solubility in an aqueous solution. In particular, compounds **7**, **8**, and **9** showed good solubility, good human intestinal absorption, and non-inhibition of the enzyme CYP2D6, as well as qualifying within the criteria of AlogP98 (<5) and PSA_2D (<140 Å2), exhibiting optimal cell permeability. Also, compounds **7**, **8**, and **9** showed >90% of plasma protein binding (PPB). Viewing the drug likeness and ADMET responses, we found that compounds **7**, **8**, and **9** are those phytochemicals that demonstrated outstanding drug-like characters and qualified for most of the ADMET standards. Though other compounds (**1**, **2**–**6**, **10**) also possess drug-like characters and qualified different parameters of ADMET, their effectiveness as drugs cannot be underestimated. Consequently, we implemented two important approaches, reversible noncovalent inhibitors as well as covalent irreversible inhibitors among compounds **1**–**10**, which can interact with 3CL^Pro^ and PL^Pro^ targets of SARS-CoV-2. In the noncovalent docking, we found that compounds **1**–**10** formed hydrogen bonds with crucial amino acid residues of 3CL^Pro^ and PL^Pro^. In the covalent docking protocol, we found that only three compounds (compounds **7**, **8**, and **9**) formed stable covalent bonding, indicating their strong binding potential via irreversible binding with the crucial amino acid residues in the active site of 3CL^Pro^ and PL^Pro^. Collectively, the non-covalent data affirmed that compounds **1**–**10** have the capability to interact with the non-crucial amino acids in the active sites of 3CL^Pro^ and PL^Pro^, while covalent docking data revealed that compounds **7**, **8**, and **9** have a surplus capability to strongly bind with crucial amino acids (CYS145 and CYS111) of 3CL^Pro^ and PL^Pro^ and act as target inhibitors. The RMSD, RMSF, and Rg data further validated the reality that the association between compounds **7**, **8**, and **9** with 3CL^Pro^, and compounds **7** and **8** with PL^Pro^ was quite stable, which did not dissociate during the MD simulations. In fact, the targets were found to be more stabilized upon their complexation with compounds **7**, **8**, and **9**. We recommend evaluating the inhibitory effects of aforementioned compounds using suitable in vitro and in vivo tests against SARS-CoV-2.

## Figures and Tables

**Table 1:** Details of phytochemicals (compounds **1**–**10**) used in the current study.

Compound Code	Compound Name	2D Structure	Name of Plants
**1**	5,3′,4′-trihydroxyflavan 7-O-gallate	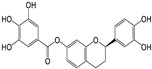	*Oncocalyx glabratus*
**2**	5,4′-dihydroxyflavan 7-3′-O-digallate	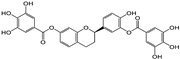	*Oncocalyx glabratus*
**3**	5,3′-dihydroxyflavan 7-4′-*O*-digallate	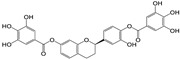	*Oncocalyx glabratus*
**4**	Spinasterol	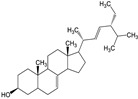	*Baccharoides* *schimperi*
**5**	Stigmasterol	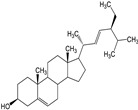	*Baccharoides* *schimperi*
**6**	3′,4′,5,7-tetrahydroxy-3-methoxyflavone	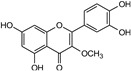	*Baccharoides* *schimperi*
**7**	Vernolepin	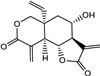	*Baccharoides* *schimperi*
**8**	Vernodalol	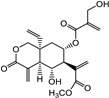	*Baccharoides* *schimperi*
**9**	11β,13-dihydrovernodalin	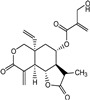	*Baccharoides* *schimperi*
**10**	Quercitrin 3-O-rhamnoside	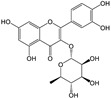	*Euphorpia schimperi*

**Table 2:** Drug likeness of compounds **1**–**10** analyzed by Veber and Lipinski’s rule of five (RO5).

Compound Code	A LogP	Mol. Wt.	nHBA	nHBD	MFPSA	Num-Ring	No. R. Bonds	TPSA	MR
**1**	4.178	410.374	8	5	0.364	4	4	136.68	105.42
**2**	5.126	562.478	12	7	0.401	5	7	203.44	140.87
**3**	5.126	562.478	12	7	0.401	5	7	203.44	140.87
**4**	7.639	412.691	1	1	0.042	4	5	20.23	128.69
**5**	7.639	412.691	1	1	0.042	4	5	20.23	128.69
**6**	1.856	316.262	7	4	0.397	3	2	120.36	82.50
**7**	0.818	276.285	5	1	0.273	3	1	72.83	66.87
**8**	0.892	392.4	8	2	0.291	2	8	119.36	95.00
**9**	1.236	362.374	7	1	0.275	3	5	99.13	86.88
**10**	0.589	448.377	11	7	0.462	4	3	190.28	103.90

**Table 3:** Compounds **1**–**10** analyzed for their predicted ADMET properties.

Compound Code	HIA Level	CYP2D6	Hepatotoxicity	PPB	Solubility Level	Molar Solubility Log(sw)	BBBP Level	AlogP98	PSA_2D
**1**	2	True	False	False	2	−5.103	4	4.178	139.238
**2**	3	True	False	False	1	−7.249	4	5.126	207.1
**3**	3	True	False	False	1	−7.127	4	5.126	207.1
**4**	3	True	False	True	1	−7.962	4	7.639	20.815
**5**	3	False	False	True	1	−7.963	4	7.639	20.815
**6**	0	True	False	False	3	−2.93	4	1.856	118.422
**7**	0	False	False	True	3	−2.392	3	0.818	73.277
**8**	0	False	False	True	3	−2.065	4	0.893	120.323
**9**	0	False	False	True	3	−2.842	3	1.236	99.508
**10**	3	False	False	False	3	−3.888	4	0.589	189.799

Aqueous Solubility Descriptors: Solubility extremely low, when level is 0 or log(sw) is <−8.0. Not soluble, very low, but possible, when level is 1 or −8.0 < log(sw) < −6.0. Solubility is yes but low, when level is 2 or −6.0 < log(sw) < −4.0. Good solubility, when level is 3 or −4.1 < log(sw) < −2.0. Solubility is optimal, when level is 4 or −2.0 < log(Sw) < 0.0. No, too soluble, when level is 5 or 0.0 < log(sw). AlogP98 (<5) good absorption through blood–brain barrier penetration level (BBB). PSA_2D. Human intestinal absorption (HIA) level: Good (0), Moderate (1), low (2), very low (3), undefined (4). Hepatotoxicity: toxic (True), non-toxic (False). Cytochrome P2D6 (CYP2D6) inhibition: non-inhibitor (False), inhibitor (True). Plasma protein binding (PPB): True = >90% binding, False = <90% binding. BBBP level: very high (0), high (1), medium (2), low (3), undefined (4) and warning, molecules with one or more unknown AlogP98 calculation (5).

## Data Availability

All data are provided in this manuscript.

## Supplementary Materials

The following supporting information can be downloaded at: https://www.mdpi.com/article/10.3390/molecules29050998/s1, Figure S1: Validation of docking protocol with 3CL^Pro^ and co-crystal ligand (X77). Figure S2: Validation of docking protocol with PL^Pro^ and co-crystal ligand (VIR250). Figures S3–S12: Surface representation showing non-covalent docking of compound **1**–**10** with target protein (3CL^Pro^). Figures S13–S22: Surface representation showing non-covalent docking of compound **1**–**10** with target protein (PL^Pro^). Figure S23: Schematic diagram of Michael addition and covalent warheads in compounds **7**–**9**. Figures S24–S26: 2D view of 3CL^Pro^ showing covalent binding with compound **7**–**9**. Figures S27–S29: 2D view of PL^Pro^ showing covalent binding with compound **7**–**9**. Figure S30: Screenshot of CHARMM-GUI exhibiting the availability of option to upload the molecule covalently bonded with the target. S31: Ligands (compounds **7**–**9**) were edited to add covalent bond with the thiol (SH) of CYS 145/111 in 3CL^Pro^ and PL^Pro^ for parameterization. S32: Screenshot from CHARMM-GUI showing the generation of PDB files that generated the topology and parameter files of the ligands. Table S1: Validation of redocking interaction between co-crystal ligand (X77) with SARS-CoV-2 target protein (3CL^Pro^). Table S2: Validation of redocking interaction between co-crystal ligand (VIR250) with SARS-CoV-2 target protein (PL^Pro^). Tables S3–S12: Non-covalent docking of compounds **1**–**10** with SARS-CoV-2 target protein (3CL^Pro^). Tables S13–S22: Non-covalent docking of compounds **1**–**10** with SARS-CoV-2 target protein (PL^Pro^). Tables S23–S25: Covalent docking of compounds **7**–**9** with SARS-CoV-2 target protein (3CL^Pro^). Tables S26–S28: Covalent docking of compounds **7**–**9** with SARS-CoV-2 target protein (PL^Pro^). Tables S29 and S30: Summarization of interaction between compounds **1**–**10** resulting in reversible (non-covalent) and irreversible (covalent) binding with 3CL^Pro^ and PL^Pro^.
