# Supplementary material for: Identification of Phytochemicals from Arabian Peninsula Medicinal Plants as Strong Binders to SARS-CoV-2 Proteases (3CLPro and PLPro) by Molecular Docking and Dynamic Simulation Studies
Source: Molecules. 2024 Feb 25;29(5):998. doi: 10.3390/molecules29050998 (PMC10934083; doi:10.3390/molecules29050998)
Supplement: Supplementary file 1 [file molecules-29-00998-s001.zip › molecules-2626825-supplementary.pdf]

Supplementary Table 1. Validation of redocking interaction between co-crystal ligand (X77) with SARS-CoV-2 target protein ($3CL^{Pro}$).

**Ligand (X77)**	**Receptor ($3CL^{Pro}$)**	**Interaction Type**	**Distance (Å)**	**Energy (kcal/mol)**	**Docking Score (kcal/mol)**
C22 17	SD MET 49 (A)	H-donor	4.07	-0.4	
N06 29	O HOH 671 (A)	H-donor	3.03	-2.4	
O01 33	CA ASN 142 (A)	H-acceptor	3.01	-1.6	
O01 33	N GLY 143 (A)	H-acceptor	2.94	-2.8	-8.3
O13 34	N GLU 166 (A)	H-acceptor	2.8	-2.3	
C26 21	5-ring HIS 41 (A)	H-π	4.31	-0.5	
6-ring	CB GLU 166 (A)	π-H	3.8	-0.5	

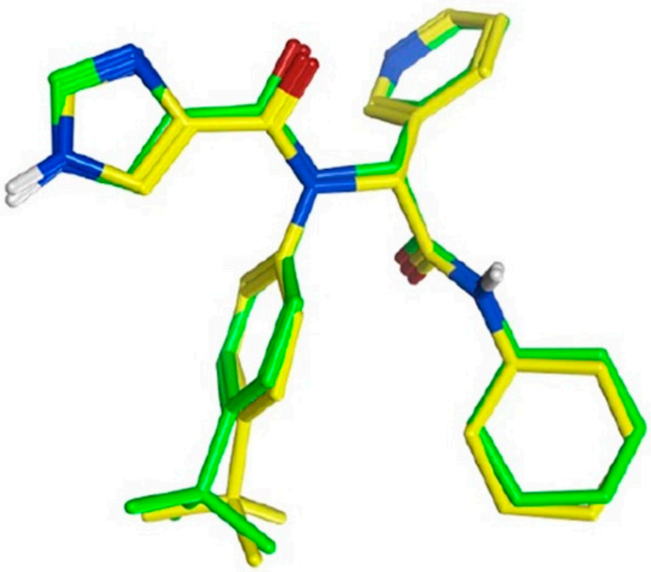

Supplementary Figure 1. Validation of docking protocol with $3CL^{Pro}$ and co-crystal ligand (X77). Superimposed image after redocking of co-crystallized ligand X77 (green) with the crystalized ligand (yellow) RSMD: 0.557 Å

Supplementary Table 2. Validation of redocking interaction between co-crystal ligand (VIR250) with SARS-CoV-2 target protein (PL^{Pro}).

Ligand (VIR250)	Receptor (PL^{Pro})	Interaction Type	Distance (Å)	Energy (kcal/mol)	Docking Score (kcal/mol)
N 32	OD1 ASP 164 (A)	H-donor	3.09	-2.4	-7.2
CB 38	OD1 ASP 164 (A)	H-donor	3.2	-1	
OE2 65	SG CYS 111 (A)	H-donor	3.07	-0.8	
O 2	NH2 ARG 166 (A)	H-acceptor	2.98	-0.5	

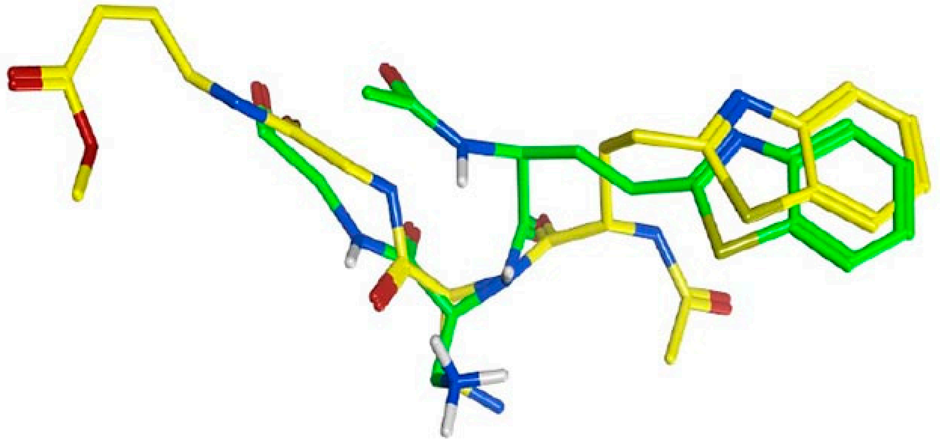

Supplementary Figure 2. Validation of docking protocol with PL^{Pro} and co-crystal ligand (VIR250). Superimposed image after redocking of co-crystallized ligand VIR250 (green) with the crystalized ligand (yellow) RSMD: 2.927 Å.

Supplementary Table 3. Non-covalent docking of 5,3′,4′-trihydroxyflavan 7-O-gallate (compound **1)** with SARS-CoV-2 target protein (3CL^{Pro}).

Ligand (Compound 1)	Receptor (3CL^{Pro})	Interaction Type	Distance (Å)	Energy (kcal/mol)	Docking Score (kcal/mol)
C 12	SD MET 165 (A)	H-donor	4.03	-0.3	-6.4
C 17	SD MET 165 (A)	H-donor	3.55	-0.3	
O 31	OD1 ASN 142 (A)	H-donor	2.97	-0.3	
O 34	O THR 26 (A)	H-donor	3.25	-1.1	
O 37	SG CYS 145 (A)	H-donor	3.75	-2.4	
6-ring	CB MET 165 (A)	π-H	3.72	-0.3	

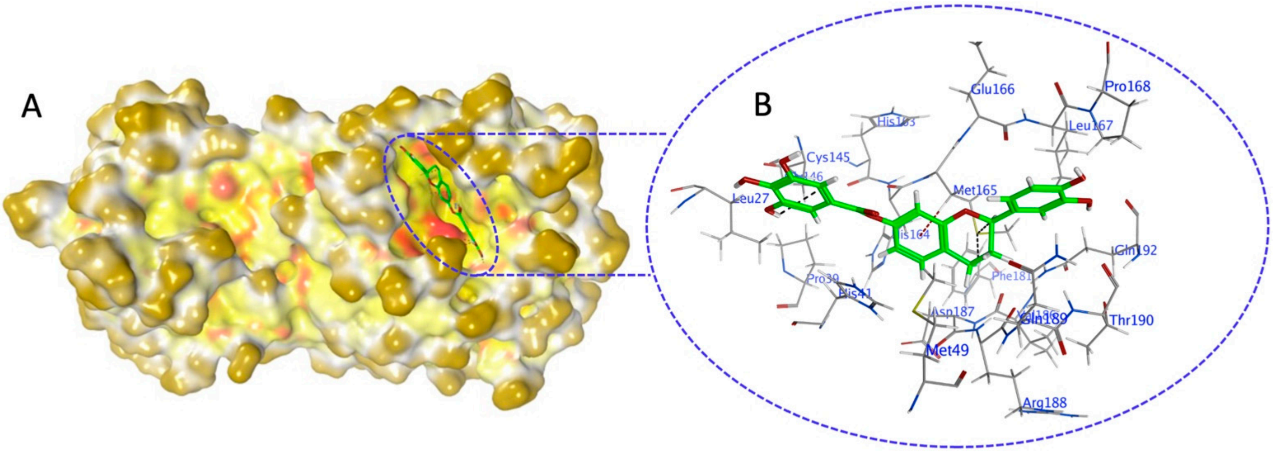

Supplementary Figure 3. Surface representation showing non-covalent docking of compound **1** with target protein (3CL^{Pro}) of SARS-CoV-2 (Panel A). Compounds **1** is in green color within the surface representation images of 3CL^{Pro}. The solvent exposed region of 3CL^{Pro} is dark yellow, hydrophobic regions are in yellow, and polar regions are in red color. (Panel B) Magnified view of 3CL^{Pro} active pocket occupied by compound **1** showing its interactions with different amino acid residues. Bond colors in the magnified view are as follows; H-bond (black color), H-π bond (dark red), Van der Waals clashes (dark blue), atoms (element color), residues are labeled as blue texts. Images were generated by using MOE software.

Supplementary Table 4. Non-covalent docking of 5,4′-dihydroxyflavan 7-3′-O-digallate (compound **2**) with SARS-CoV-2 target protein ($3CL^{Pro}$).

Ligand (Compound 2)	Receptor ($3CL^{Pro}$)	Interaction Type	Distance (Å)	Energy (kcal/mol)	Docking Score (kcal/mol)
O 58	SG CYS 145 (A)	H-donor	3.27	-3.9	
O 58	N GLY 143 (A)	H-acceptor	3.05	-0.7	-7.4
6-ring	CB MET 165 (A)	π-H	3.7	-0.6	

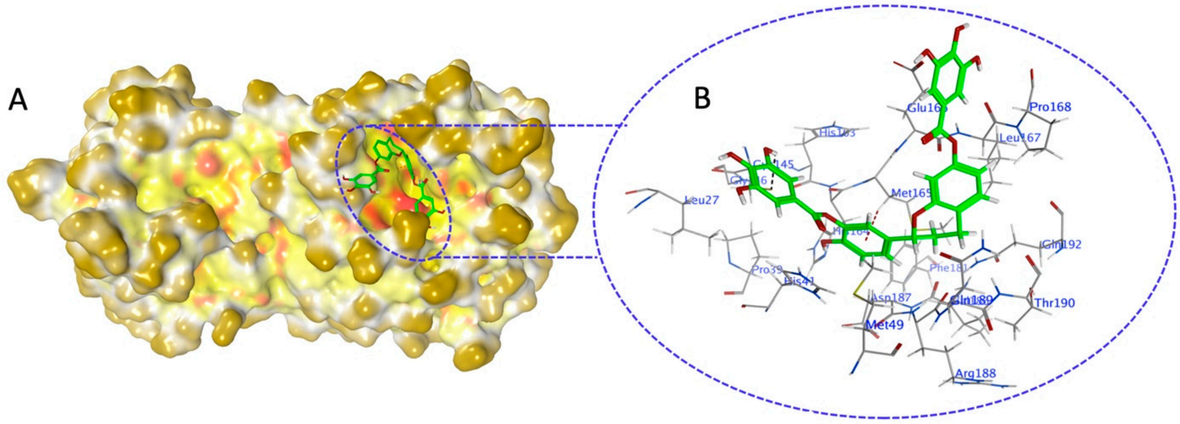

Supplementary Figure 4. Surface representation showing non-covalent docking of compound **2** with target protein ($3CL^{Pro}$) of SARS-CoV-2 (Panel A). Compounds **2** is in green color within the surface representation images of $3CL^{Pro}$. The solvent exposed region of $3CL^{Pro}$ is dark yellow, hydrophobic regions are in yellow, and polar regions are in red color. (Panel B) Magnified view of $3CL^{Pro}$ active pocket occupied by compound **2** showing its interactions with different amino acid residues. Bond colors in the magnified view are as follows; H-bond (black color), H-π bond (dark red), Van der Waals clashes (dark blue), atoms (element color), residues are labeled as blue texts. Images were generated by using MOE software.

Supplementary Table 5. Non-covalent docking of 5,3′-dihydroxyflavan 7-4′-O-digallate (compound **3**) with SARS-CoV-2 target protein ($3CL^{Pro}$).

Ligand (Compound 3)	Receptor ($3CL^{Pro}$)	Interaction Type	Distance (Å)	Energy (kcal/mol)	Docking Score (kcal/mol)
C 7	SG CYS 145 (A)	H-donor	3.75	-2.5	
O 10	SG CYS 145 (A)	H-donor	4.38	-3.7	
C 19	SG CYS 145 (A)	H-donor	3.58	-0.4	
C 24	SD MET 165 (A)	H-donor	3.64	-0.3	
C 30	SD MET 165 (A)	H-donor	3.81	-0.3	
O 16	NE2 HIS 41 (A)	H-acceptor	2.98	-0.7	-7.8
6-ring	CG2 THR 25 (A)	π-H	4.22	-0.7	
6-ring	CB MET 165 (A)	π-H	4.47	-0.3	
6-ring	CA GLN 189 (A)	π-H	4.75	-0.6	
6-ring	5-ring HIS 41 (A)	π-π	3.48	0	

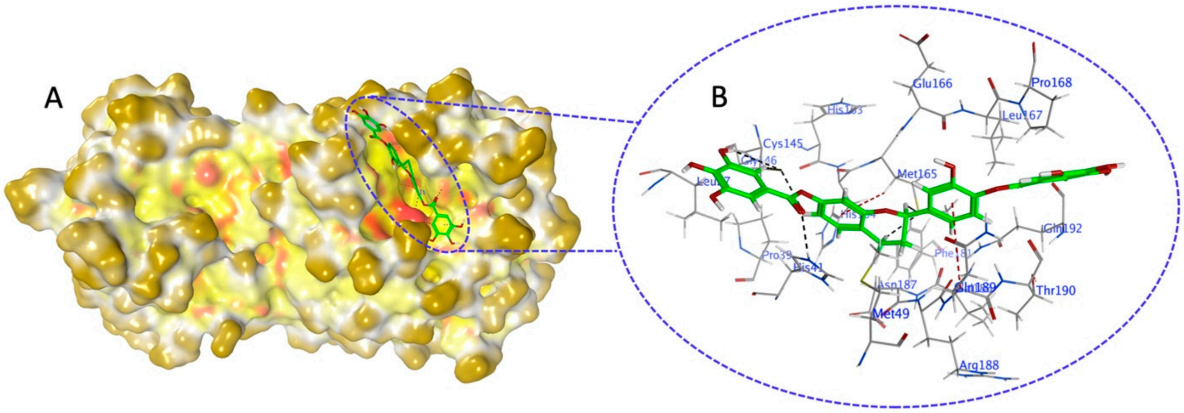

Supplementary Figure 5. Surface representation showing non-covalent docking of compound **3** with target protein ($3CL^{Pro}$) of SARS-CoV-2 (Panel A). Compounds **3** is in green color within the surface representation images of $3CL^{Pro}$. The solvent exposed region of $3CL^{Pro}$ is dark yellow, hydrophobic regions are in yellow, and polar regions are in red color. (Panel B) Magnified view of $3CL^{Pro}$ active pocket occupied by compound **3** showing its interactions with different amino acid residues. Bond colors in the magnified view are as follows; H-bond (black color), H-π bond (dark red), Van der Waals clashes (dark blue), atoms (element color), residues are labeled as blue texts. Images were generated by using MOE software.

Supplementary Table 6. Non-covalent docking of spinasterol (compound **4**) with SARS-CoV-2 target protein (3CL^{Pro}).

Ligand (Compound 4)	Receptor (3CL^{Pro})	Interaction Type	Distance (Å)	Energy (kcal/mol)	Docking Score (kcal/mol)
O 1	O THR 190 (A)	H-donor	2.74	-1.5	
C 22	SD MET 165 (A)	H-donor	4.2	-0.2	
C 60	SG CYS 145 (A)	H-donor	4.06	-0.3	-6.6
O 1	NE2 GLN 192 (A)	H-acceptor	3.23	-1.2	
C 27	5-ring HIS 41 (A)	H-π	4.05	-0.5	

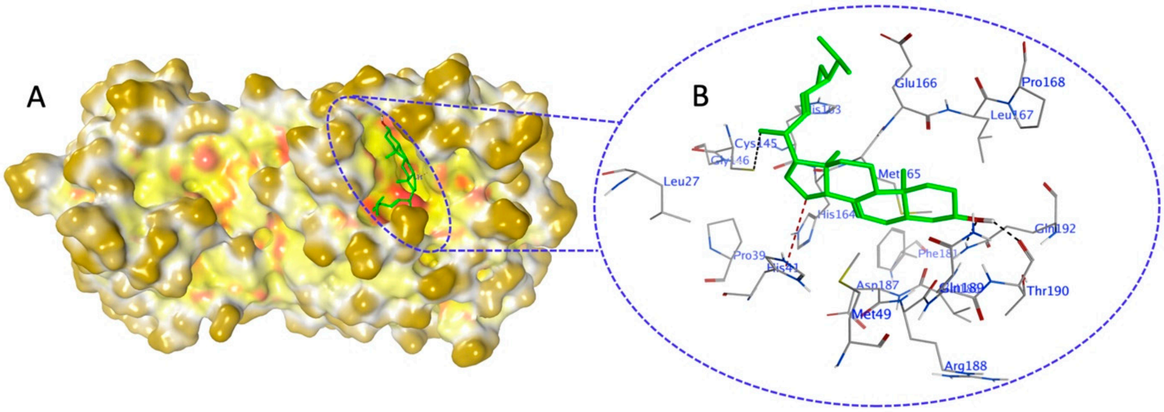

Supplementary Figure 6. Surface representation showing non-covalent docking of compound **4** with target protein (3CL^{Pro}) of SARS-CoV-2 (Panel A). Compounds **4** is in green color within the surface representation images of 3CL^{Pro}. The solvent exposed region of 3CL^{Pro} is dark yellow, hydrophobic regions are in yellow, and polar regions are in red color. (Panel B) Magnified view of 3CL^{Pro} active pocket occupied by compound **4** showing its interactions with different amino acid residues. Bond colors in the magnified view are as follows; H-bond (black color), H-π bond (dark red), Van der Waals clashes (dark blue), atoms (element color), residues are labeled as blue texts. Images were generated by using MOE software.

Supplementary Table 7. Non-covalent docking of stigmasterol (compound **5**) with SARS-CoV-2 target protein (3CL^{Pro}).

Ligand (Compound 5)	Receptor (3CL^{Pro})	Interaction Type	Distance (Å)	Energy (kcal/mol)	Docking Score (kcal/mol)
C 1	SG CYS 145 (A)	H-donor	3.92	-0.2	-6.3
C 5	SG CYS 145 (A)	H-donor	4.21	-0.2	
C 22	SG CYS 145 (A)	H-donor	4.06	-0.2	
C 25	SG CYS 145 (A)	H-donor	3.77	-0.2	
C 35	SD MET 165 (A)	H-donor	3.7	-0.2	
O 43	O THR 190 (A)	H-donor	3.41	-0.2	
O 43	NE2 GLN 192 (A)	H-acceptor	3.16	-1.7	
C 32	5-ring HIS 41 (A)	H-π	4.21	-0.3	

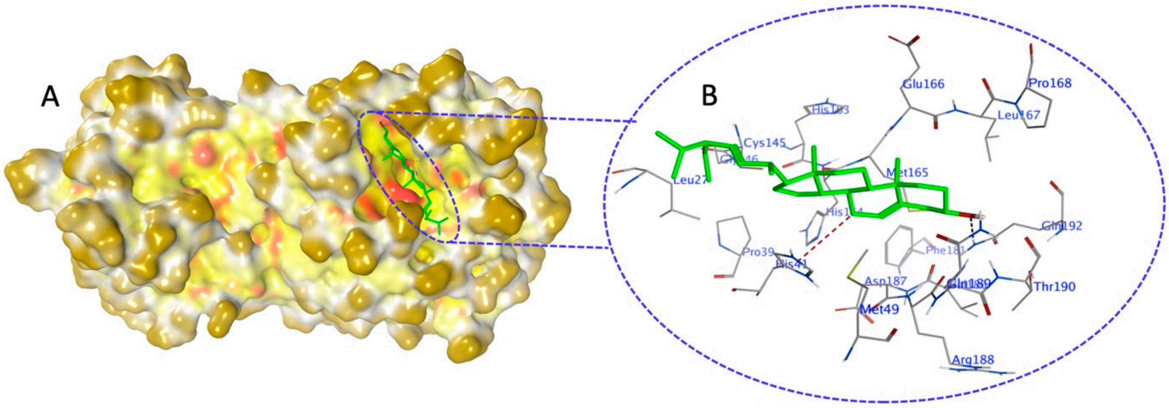

Supplementary Figure 7. Surface representation showing non-covalent docking of compound **5** with target protein (3CL^{Pro}) of SARS-CoV-2 (Panel A). Compounds **5** is in green color within the surface representation images of 3CL^{Pro}. The solvent exposed region of 3CL^{Pro} is dark yellow, hydrophobic regions are in yellow, and polar regions are in red color. (Panel B) Magnified view of 3CL^{Pro} active pocket occupied by compound **5** showing its interactions with different amino acid residues. Bond colors in the magnified view are as follows; H-bond (black color), H-π bond (dark red), Van der Waals clashes (dark blue), atoms (element color), residues are labeled as blue texts. Images were generated by using MOE software.

Supplementary Table 8. Non-covalent docking of 3′,4′,5,7-tetrahydroxy-3-methoxyflavone (compound **6**) with SARS-CoV-2 target protein ($3CL^{Pro}$).

Ligand (Compound 6)	Receptor ($3CL^{Pro}$)	Interaction Type	Distance (Å)	Energy (kcal/mol)	Docking Score (kcal/mol)
O 19	O THR 190 (A)	H-donor	2.8	-1.5	-6.2
O 27	SG CYS 145 (A)	H-donor	3.12	-8.7	
C 29	SG CYS 145 (A)	H-donor	3.86	-0.3	
6-ring	5-ring HIS 41 (A)	π-π	3.63	0	

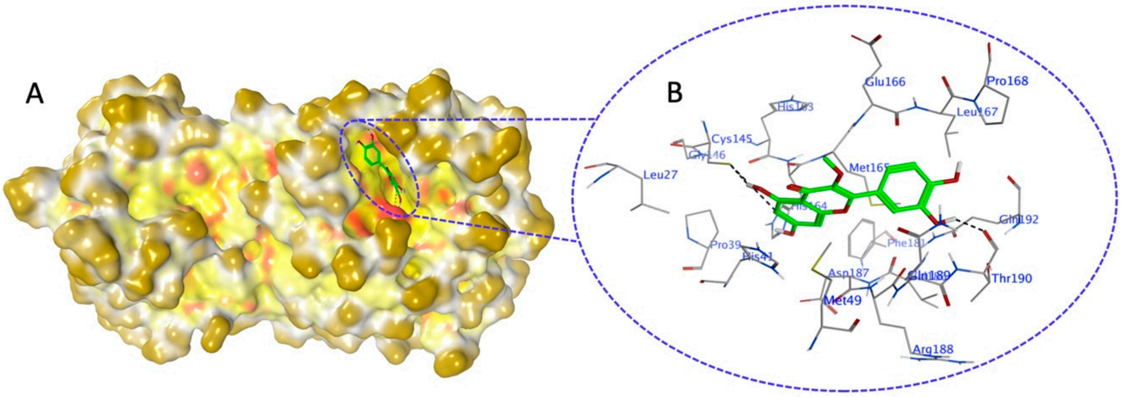

Supplementary Figure 8. Surface representation showing non-covalent docking of compound **6** with target protein ($3CL^{Pro}$) of SARS-CoV-2 (Panel A). Compounds **6** is in green color within the surface representation images of $3CL^{Pro}$. The solvent exposed region of $3CL^{Pro}$ is dark yellow, hydrophobic regions are in yellow, and polar regions are in red color. (Panel B) Magnified view of $3CL^{Pro}$ active pocket occupied by compound **6** showing its interactions with different amino acid residues. Bond colors in the magnified view are as follows; H-bond (black color), H-π bond (dark red), Van der Waals clashes (dark blue), atoms (element color), residues are labeled as blue texts. Images were generated by using MOE software.

Supplementary Table 9. Non-covalent docking of vernolepin (compound **7**) with SARS-CoV-2 target protein ($3CL^{Pro}$).

Ligand (Compound 7)	Receptor ($3CL^{Pro}$)	Interaction Type	Distance (Å)	Energy (kcal/mol)	Docking Score (kcal/mol)
C 14	SG CYS 145 (A)	H-donor	3.56	-0.8	-4.2

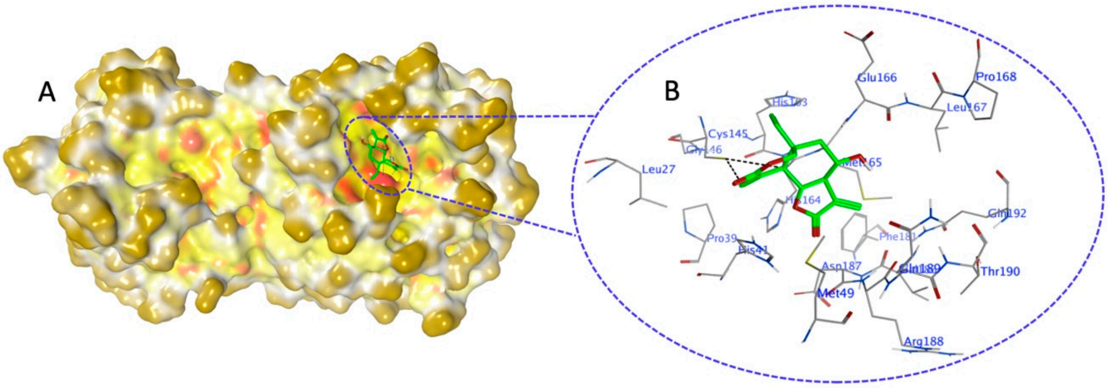

Supplementary Figure 9. Surface representation showing non-covalent docking of compound **7** with target protein ($3CL^{Pro}$) of SARS-CoV-2 (Panel A). Compounds **7** is in green color within the surface representation images of $3CL^{Pro}$. The solvent exposed region of $3CL^{Pro}$ is dark yellow, hydrophobic regions are in yellow, and polar regions are in red color. (Panel B) Magnified view of $3CL^{Pro}$ active pocket occupied by compound **7** showing its interactions with different amino acid residues. Bond colors in the magnified view are as follows; H-bond (black color), H-π bond (dark red), Van der Waals clashes (dark blue), atoms (element color), residues are labeled as blue texts. Images were generated by using MOE software.

Supplementary Table 10. Non-covalent docking of Vernadalol (compound **8**) with SARS-CoV-2 target protein (3CL^{Pro}).

Ligand (Compound 8)	Receptor (3CL^{Pro})	Interaction Type	Distance (Å)	Energy (kcal/mol)	Docking Score (kcal/mol)
O 21	SG CYS 145 (A)	H-donor	3.43	-3.1	-6.0
O 48	OE1 GLN 189 (A)	H-donor	2.97	-0.5	
O 12	N GLY 143 (A)	H-acceptor	3.41	-1.1	
O 48	N THR 190 (A)	H-acceptor	3.5	-0.4	

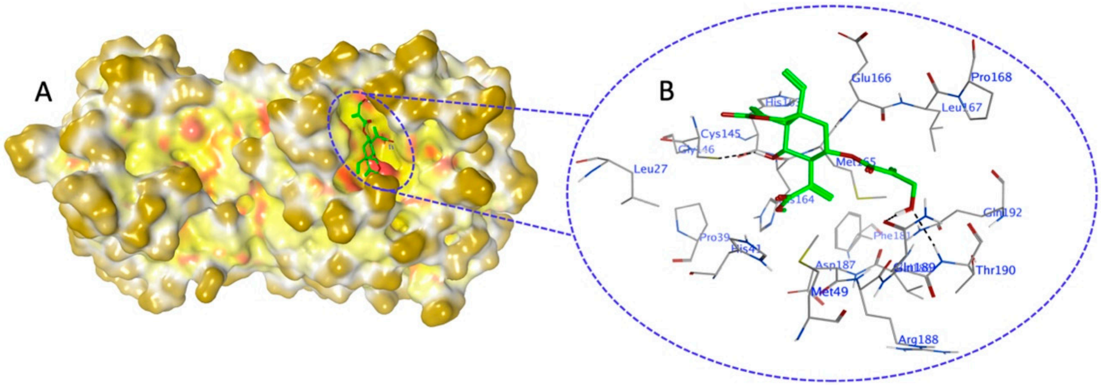

Supplementary Figure 10. Surface representation showing non-covalent docking of compound **8** with target protein (3CL^{Pro}) of SARS-CoV-2 (Panel A). Compounds **8** is in green color within the surface representation images of 3CL^{Pro}. The solvent exposed region of 3CL^{Pro} is dark yellow, hydrophobic regions are in yellow, and polar regions are in red color. (Panel B) Magnified view of 3CL^{Pro} active pocket occupied by compound **8** showing its interactions with different amino acid residues. Bond colors in the magnified view are as follows; H-bond (black color), H-π bond (dark red), Van der Waals clashes (dark blue), atoms (element color), residues are labeled as blue texts. Images were generated by using MOE software.

Supplementary Table 11. Non-covalent docking of 11β,13-dihydrovernodalin (compound **9**) with SARS-CoV-2 target protein (3CL^{Pro}).

Ligand (Compound 9)	Receptor (3CL^{Pro})	Interaction Type	Distance (Å)	Energy (kcal/mol)	Docking Score (kcal/mol)
O 7	O THR 190 (A)	H-donor	2.85	-1.9	-5.8
C 21	SG CYS 145 (A)	H-donor	3.56	-1.1	
C 25	O ARG 188 (A)	H-donor	3.52	-0.3	
O 7	NE2 GLN 192 (A)	H-acceptor	3.34	-0.6	

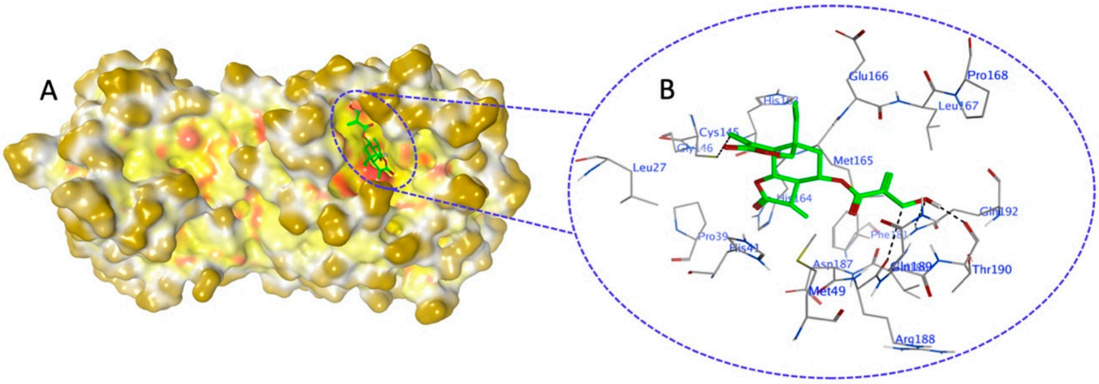

Supplementary Figure 11. Surface representation showing non-covalent docking of compound **9** with target protein (3CL^{Pro}) of SARS-CoV-2 (Panel A). Compounds **9** is in green color within the surface representation images of 3CL^{Pro}. The solvent exposed region of 3CL^{Pro} is dark yellow, hydrophobic regions are in yellow, and polar regions are in red color. (Panel B) Magnified view of 3CL^{Pro} active pocket occupied by compound **9** showing its interactions with different amino acid residues. Bond colors in the magnified view are as follows; H-bond (black color), H-π bond (dark red), Van der Waals clashes (dark blue), atoms (element color), residues are labeled as blue texts. Images were generated by using MOE software.

Supplementary Table 12. Non-covalent docking of Quercitrin 3-O-rhamnoside (compound **10**) with SARS-CoV-2 target protein (3CL^{Pro}).

Ligand (Compound 10)	Receptor (3CL^{Pro})	Interaction Type	Distance (Å)	Energy (kcal/mol)	Docking Score (kcal/mol)
C 5	SG CYS 145 (A)	H-donor	3.47	-1.8	-7.6
O 21	O THR 190 (A)	H-donor	2.83	-0.6	
O 34	ND1 HIS 164 (A)	H-donor	2.87	-1.7	
O 37	SG CYS 145 (A)	H-donor	2.88	-8	
C 45	SG CYS 145 (A)	H-donor	4.49	-0.9	
O 21	NE2 GLN 192 (A)	H-acceptor	3.17	-0.3	
6-ring	CB MET 165 (A)	π-H	4.06	-0.3	
6-ring	5-ring HIS 41 (A)	π-π	3.71	0	

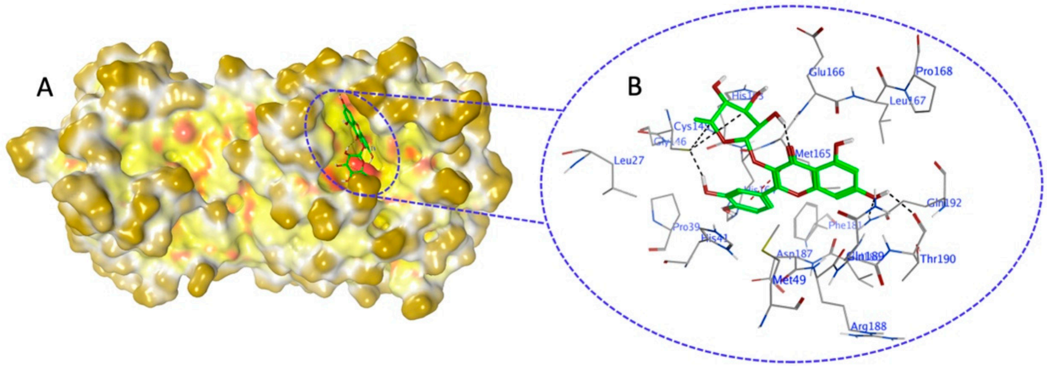

Supplementary Figure 12. Surface representation showing non-covalent docking of compound **10** with target protein (3CL^{Pro}) of SARS-CoV-2 (Panel A). Compounds **10** is in green color within the surface representation images of 3CL^{Pro}. The solvent exposed region of 3CL^{Pro} is dark yellow, hydrophobic regions are in yellow, and polar regions are in red color. (Panel B) Magnified view of 3CL^{Pro} active pocket occupied by compound **10** showing its interactions with different amino acid residues. Bond colors in the magnified view are as follows; H-bond (black color), H-π bond (dark red), Van der Waals clashes (dark blue), atoms (element color), residues are labeled as blue texts. Images were generated by using MOE software.

Supplementary Table 13. Non-covalent docking of 5,3′,4′-trihydroxyflavan 7-O-gallate (compound **1**) with SARS-CoV-2 target protein (PL^{Pro}).

Ligand (Compound 1)	Receptor (PL^{Pro})	Interaction Type	Distance (Å)	Energy (kcal/mol)	Docking Score (kcal/mol)
O 5	OG1 THR 301 (B)	H-donor	3.04	-1.1	
C 17	O TYR 268 (B)	H-donor	3.22	-0.2	
C 28	SG CYS 270 (B)	H-donor	3.78	-0.4	-5.9
O 31	SG CYS 270 (B)	H-donor	3.19	-2.3	
O 37	SG CYS 111 (B)	H-donor	3.7	-0.2	
O 41	SG CYS 111 (B)	H-donor	3.55	-0.2	
O 41	N GLY 163 (B)	H-acceptor	3.64	-0.3	
6-ring	CB LEU 162 (B)	π-H	3.99	-0.3	

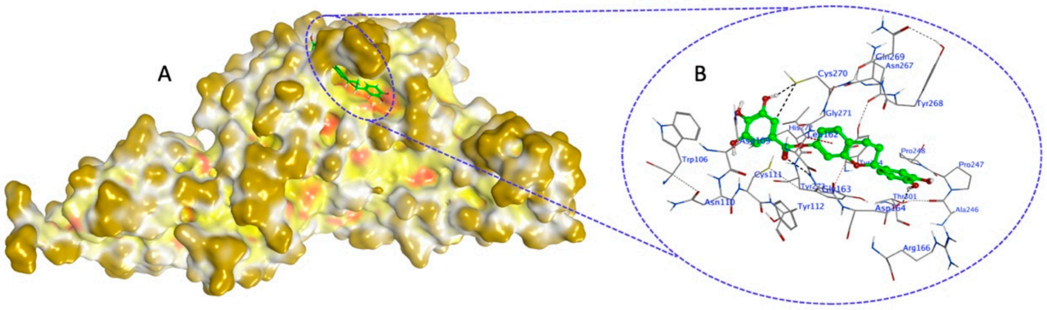

Supplementary Figure 13. Surface representation showing non-covalent docking of compound **1** with target protein (PL^{Pro}) of SARS-CoV-2 (Panel A). Compounds **1** is in green color within the surface representation images of PL^{Pro}. The solvent exposed region of PL^{Pro} is dark yellow, hydrophobic regions are in yellow, and polar regions are in red color. (Panel B) Magnified view of PL^{Pro} active pocket occupied by compound **1** showing its interactions with different amino acid residues. Bond colors in the magnified view are as follows; H-bond (black color), H-π bond (dark red), Van der Waals clashes (dark blue), atoms (element color), residues are labeled as blue texts. Images were generated by using MOE software.

Supplementary Table 14. Non-covalent docking of 5,4′-dihydroxyflavan 7-3′-O-digallate (compound **2**) with SARS-CoV-2 target protein (PL^{Pro}).

Ligand (Compound 2)	Receptor (PL^{Pro})	Interaction Type	Distance (Å)	Energy (kcal/mol)	Docking Score (kcal/mol)
O 16	SG CYS 111 (B)	H-donor	2.88	-1.8	-5.4
C 34	SG CYS 270 (B)	H-donor	3.71	-0.4	
C 24	6-ring TRP 106 (B)	H-π	4.31	-0.3	
6-ring	CB LEU 162 (B)	π-H	4.13	-0.3	

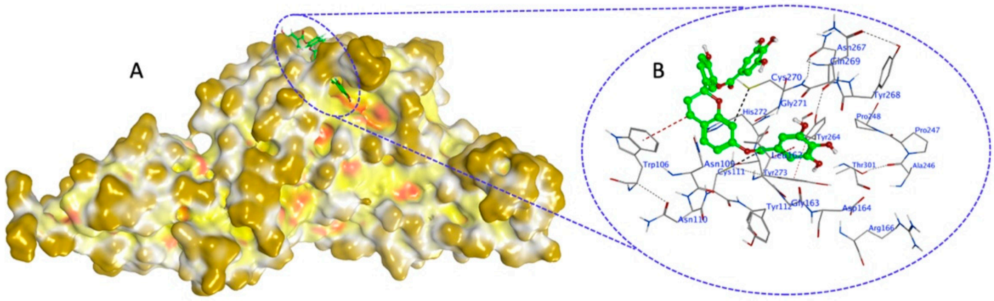

Supplementary Figure 14. Surface representation showing non-covalent docking of compound **2** with target protein (PL^{Pro}) of SARS-CoV-2 (Panel A). Compounds **2** is in green color within the surface representation images of PL^{Pro}. The solvent exposed region of PL^{Pro} is dark yellow, hydrophobic regions are in yellow, and polar regions are in red color. (Panel B) Magnified view of PL^{Pro} active pocket occupied by compound **2** showing its interactions with different amino acid residues. Bond colors in the magnified view are as follows; H-bond (black color), H-π bond (dark red), Van der Waals clashes (dark blue), atoms (element color), residues are labeled as blue texts. Images were generated by using MOE software.

Supplementary Table 15. Non-covalent docking of 5,3′-dihydroxyflavan 7-4′-O-digallate (compound **3**) with SARS-CoV-2 target protein (PL^{Pro}).

Ligand (Compound 3)	Receptor (PL^{Pro})	Interaction Type	Distance (Å)	Energy (kcal/mol)	Docking Score (kcal/mol)
O 10	O GLN 269 (B)	H-donor	2.81	-0.2	-5.0
O 13	O TYR 268 (B)	H-donor	3.11	-1.5	
O 16	SG CYS 111 (B)	H-donor	3.03	-1.1	
O 17	SG CYS 111 (B)	H-donor	3.66	-0.2	
C 34	SG CYS 270 (B)	H-donor	3.82	-0.6	
O 52	O CYS 270 (B)	H-donor	2.94	-2.3	
C 27	5-ring HIS 272 (B)	H-π	4.43	-0.3	
O 60	5-ring HIS 272 (B)	H-π	4.61	-0.4	
C 62	5-ring HIS 272 (B)	H-π	4.35	-0.9	
6-ring	CB LEU 162 (B)	π-H	4.09	-0.2	

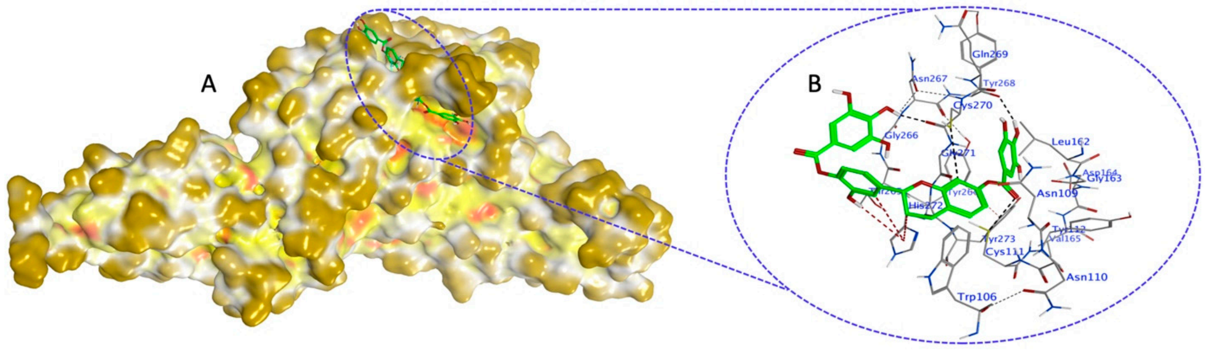

Supplementary Figure 15. Surface representation showing non-covalent docking of compound **3** with target protein (PL^{Pro}) of SARS-CoV-2 (Panel A). Compounds **3** is in green color within the surface representation images of PL^{Pro}. The solvent exposed region of PL^{Pro} is dark yellow, hydrophobic regions are in yellow, and polar regions are in red color. (Panel B) Magnified view of PL^{Pro} active pocket occupied by compound **3** showing its interactions with different amino acid residues. Bond colors in the magnified view are as follows; H-bond (black color), H-π bond (dark red), Van der Waals clashes (dark blue), atoms (element color), residues are labeled as blue texts. Images were generated by using MOE software.

Supplementary Table 16. Non-covalent docking of Spinasterol (compound **4**) with SARS-CoV-2 target protein (PL^{Pro}).

Ligand (Compound 4)	Receptor (PL^{Pro})	Interaction Type	Distance (Å)	Energy (kcal/mol)	Docking Score (kcal/mol)
C 65	SG CYS 270 (B)	H-donor	3.83	-0.2	-4.3
C 75	ND1 HIS 272 (B)	H-donor	3.86	-0.2	
C 17	5-ring HIS 272 (B)	H-π	4.4	-0.4	
C 22	5-ring HIS 272 (B)	H-π	4.11	-0.4	

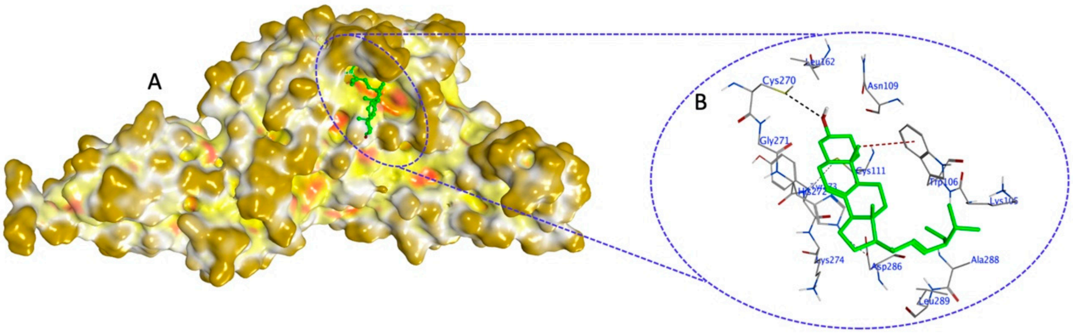

Supplementary Figure 16. Surface representation showing non-covalent docking of compound **4** with target protein (PL^{Pro}) of SARS-CoV-2 (Panel A). Compounds **4** is in green color within the surface representation images of PL^{Pro}. The solvent exposed region of PL^{Pro} is dark yellow, hydrophobic regions are in yellow, and polar regions are in red color. (Panel B) Magnified view of PL^{Pro} active pocket occupied by compound **4** showing its interactions with different amino acid residues. Bond colors in the magnified view are as follows; H-bond (black color), H-π bond (dark red), Van der Waals clashes (dark blue), atoms (element color), residues are labeled as blue texts. Images were generated by using MOE software.

Supplementary Table 17. Non-covalent docking of Stigmasterol (compound **5**) with SARS-CoV-2 target protein (PL^{Pro}).

Ligand (Compound 5)	Receptor (PL^{Pro})	Interaction Type	Distance (Å)	Energy (kcal/mol)	Docking Score (kcal/mol)
C 10	SG CYS 270 (B)	H-donor	3.83	-0.2	-5.2
C 41	OD2 ASP 286 (B)	H-donor	3.17	-0.2	
C 52	6-ring TRP 106 (B)	H-π	4.4	-0.2	

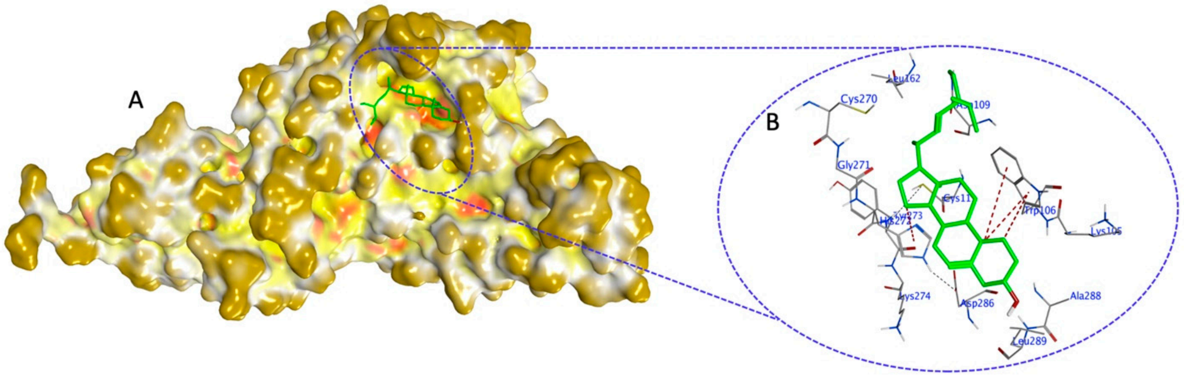

Supplementary Figure 17. Surface representation showing non-covalent docking of compound **5** with target protein (PL^{Pro}) of SARS-CoV-2 (Panel A). Compounds **5** is in green color within the surface representation images of PL^{Pro}. The solvent exposed region of PL^{Pro} is dark yellow, hydrophobic regions are in yellow, and polar regions are in red color. (Panel B) Magnified view of PL^{Pro} active pocket occupied by compound **5** showing its interactions with different amino acid residues. Bond colors in the magnified view are as follows; H-bond (black color), H-π bond (dark red), Van der Waals clashes (dark blue), atoms (element color), residues are labeled as blue texts. Images were generated by using MOE software.

Supplementary Table 18. Non-covalent docking of 3′,4′,5,7-tetrahydroxy-3-methoxyflavone (compound **6**) with SARS-CoV-2 target protein (PL^{Pro}).

Ligand (Compound 6)	Receptor (PL^{Pro})	Interaction Type	Distance (Å)	Energy (kcal/mol)	Docking Score (kcal/mol)
O 19	O CYS 270 (B)	H-donor	2.85	-2	-4.7
C 34	SG CYS 270 (B)	H-donor	4.12	-0.5	
C 5	5-ring HIS 272 (B)	H-π	4.08	-1.2	
6-ring	CB HIS 272 (B)	π-H	3.95	-0.4	

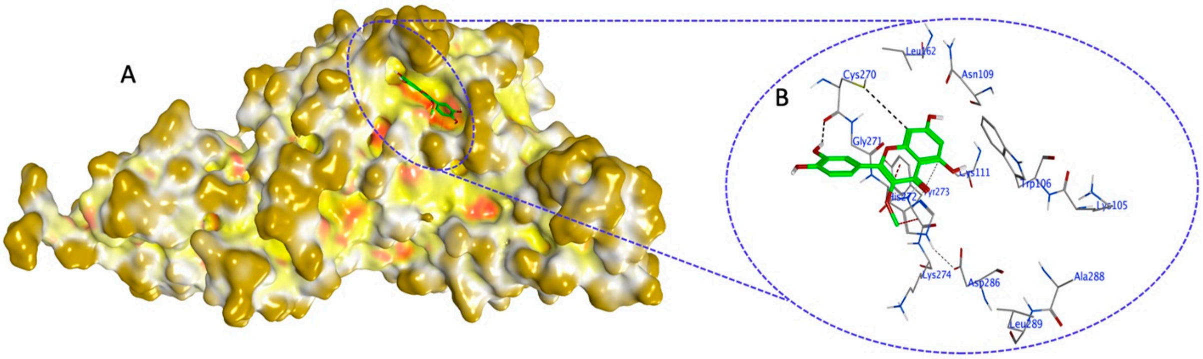

Supplementary Figure 18. Surface representation showing non-covalent docking of compound **6** with target protein (PL^{Pro}) of SARS-CoV-2 (Panel A). Compounds **6** is in green color within the surface representation images of PL^{Pro}. The solvent exposed region of PL^{Pro} is dark yellow, hydrophobic regions are in yellow, and polar regions are in red color. (Panel B) Magnified view of PL^{Pro} active pocket occupied by compound **6** showing its interactions with different amino acid residues. Bond colors in the magnified view are as follows; H-bond (black color), H-π bond (dark red), Van der Waals clashes (dark blue), atoms (element color), residues are labeled as blue texts. Images were generated by using MOE software.

Supplementary Table 19. Non-covalent docking of Vernolepin (compound **7**) with SARS-CoV-2 target protein (PL^{Pro}).

Ligand (Compound 7)	Receptor (PL^{Pro})	Interaction Type	Distance (Å)	Energy (kcal/mol)	Docking Score (kcal/mol)
O 23	SG CYS 111 (B)	H-donor	3.26	-0.2	
O 32	SG CYS 270 (B)	H-donor	3.3	-0.4	
O 32	O GLY 271 (B)	H-donor	2.99	-0.7	-4.8
O 23	CA ASN 109 (B)	H-acceptor	3.46	-0.3	
C 14	6-ring TRP 106 (B)	H-π	3.57	-0.7	
C 17	5-ring HIS 272 (B)	H-π	4.21	-0.3	

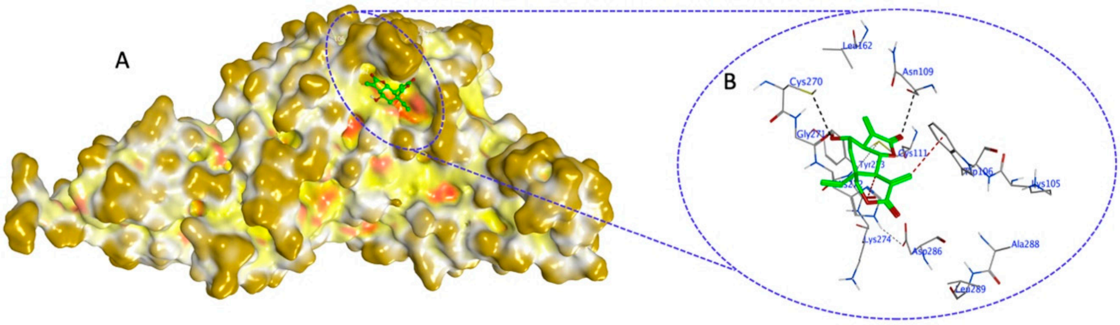

Supplementary Figure 19. Surface representation showing non-covalent docking of compound **7** with target protein (PL^{Pro}) of SARS-CoV-2 (Panel A). Compounds **7** is in green color within the surface representation images of PL^{Pro}. The solvent exposed region of PL^{Pro} is dark yellow, hydrophobic regions are in yellow, and polar regions are in red color. (Panel B) Magnified view of PL^{Pro} active pocket occupied by compound **7** showing its interactions with different amino acid residues. Bond colors in the magnified view are as follows; H-bond (black color), H-π bond (dark red), Van der Waals clashes (dark blue), atoms (element color), residues are labeled as blue texts. Images were generated by using MOE software.

Supplementary Table 20. Non-covalent docking of Vernodalol (compound **8**) with SARS-CoV-2 target protein (PL^{Pro}).

Ligand (Compound 8)	Receptor (PL^{Pro})	Interaction Type	Distance (Å)	Energy (kcal/mol)	Docking Score (kcal/mol)
C 7	O TYR 268 (B)	H-donor	3.43	-0.2	
C 36	O TYR 268 (B)	H-donor	3.64	-0.3	-5.4
O 48	SG CYS 111 (B)	H-donor	3.7	-0.9	

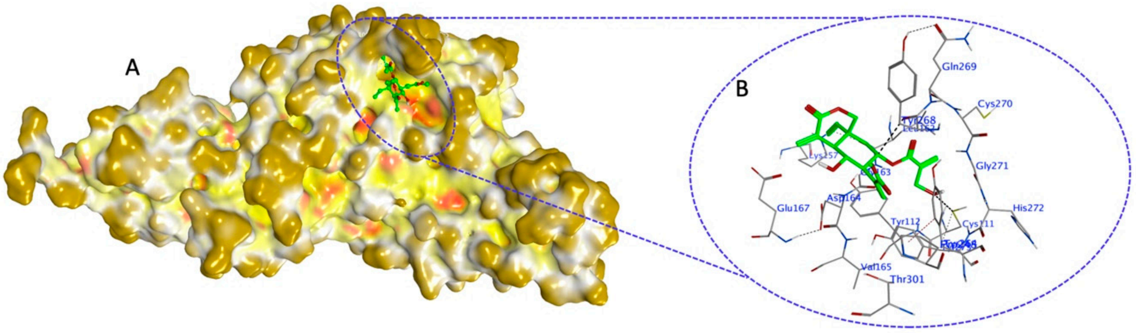

Supplementary Figure 20. Surface representation showing non-covalent docking of compound **8** with target protein (PL^{Pro}) of SARS-CoV-2 (Panel A). Compounds **8** is in green color within the surface representation images of PL^{Pro}. The solvent exposed region of PL^{Pro} is dark yellow, hydrophobic regions are in yellow, and polar regions are in red color. (Panel B) Magnified view of PL^{Pro} active pocket occupied by compound **8** showing its interactions with different amino acid residues. Bond colors in the magnified view are as follows; H-bond (black color), H-π bond (dark red), Van der Waals clashes (dark blue), atoms (element color), residues are labeled as blue texts. Images were generated by using MOE software.

Supplementary Table 21. Non-covalent docking of 11β,13-dihydrovernodalin (compound **9**) with SARS-CoV-2 target protein (PL^{Pro}).

Ligand (Compound 9)	Receptor (PL^{Pro})	Interaction Type	Distance (Å)	Energy (kcal/mol)	Docking Score (kcal/mol)
O 7	SG CYS 111 (B)	H-donor	3.75	-0.5	
C 11	O TYR 268 (B)	H-donor	3.62	-0.4	
C 15	O TYR 268 (B)	H-donor	3.34	-0.8	
C 25	SG CYS 111 (B)	H-donor	3.77	-0.2	-5.9
C 26	SG CYS 111 (B)	H-donor	3.57	-0.3	
O 6	N GLY 271 (B)	H-acceptor	2.96	-1.6	
O 7	N GLY 163 (B)	H-acceptor	3.05	-1.8	

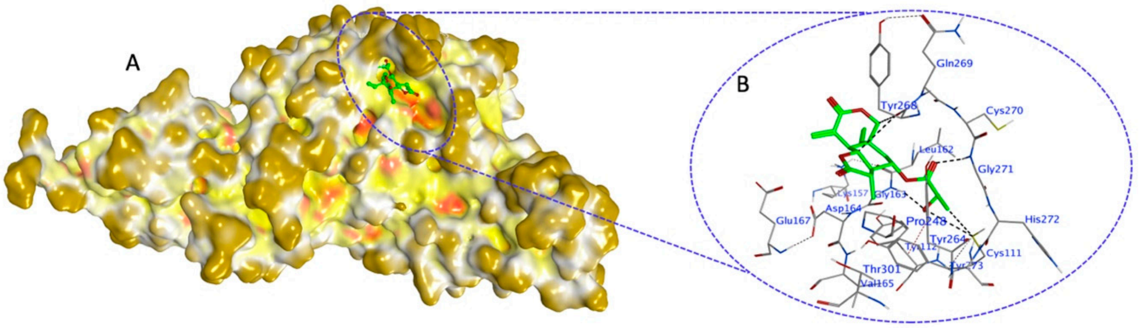

Supplementary Figure 21. Surface representation showing non-covalent docking of compound **9** with target protein (PL^{Pro}) of SARS-CoV-2 (Panel A). Compounds **9** is in green color within the surface representation images of PL^{Pro}. The solvent exposed region of PL^{Pro} is dark yellow, hydrophobic regions are in yellow, and polar regions are in red color. (Panel B) Magnified view of PL^{Pro} active pocket occupied by compound **9** showing its interactions with different amino acid residues. Bond colors in the magnified view are as follows; H-bond (black color), H-π bond (dark red), Van der Waals clashes (dark blue), atoms (element color), residues are labeled as blue texts. Images were generated by using MOE software.

Supplementary Table 22. Non-covalent docking of Quercitrin 3-O-rhamnoside (compound **10**) with SARS-CoV-2 target protein (PL^{Pro}).

Ligand (Compound 10)	Receptor (PL^{Pro})	Interaction Type	Distance (Å)	Energy (kcal/mol)	Docking Score (kcal/mol)
O 16	OE2 GLU 167 (B)	H-donor	3.25	-0.3	-5.8
C 45	O TYR 268 (B)	H-donor	3.34	-0.2	
O 51	O GLN 269 (B)	H-donor	2.9	-0.6	
C 31	6-ring TYR 264 (B)	H-π	4.07	-0.3	

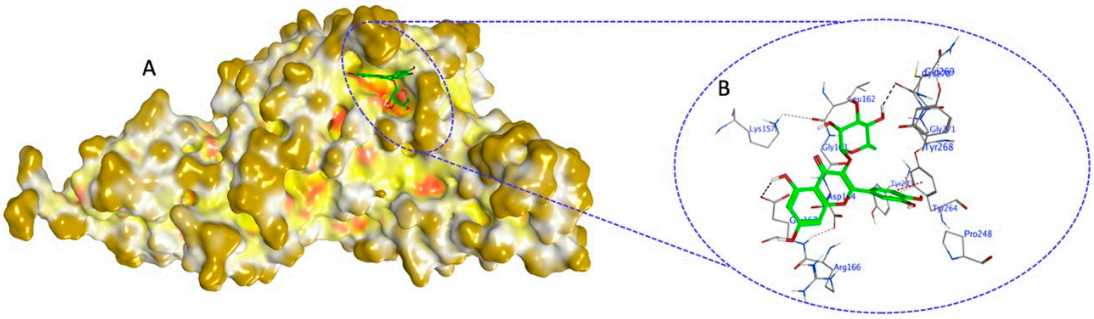

Supplementary Figure 22. Surface representation showing non-covalent docking of compound **10** with target protein (PL^{Pro}) of SARS-CoV-2 (Panel A). Compounds **10** is in green color within the surface representation images of PL^{Pro}. The solvent exposed region of PL^{Pro} is dark yellow, hydrophobic regions are in yellow, and polar regions are in red color. (Panel B) Magnified view of PL^{Pro} active pocket occupied by compound **10** showing its interactions with different amino acid residues. Bond colors in the magnified view are as follows; H-bond (black color), H-π bond (dark red), Van der Waals clashes (dark blue), atoms (element color), residues are labeled as blue texts. Images were generated by using MOE software.

A

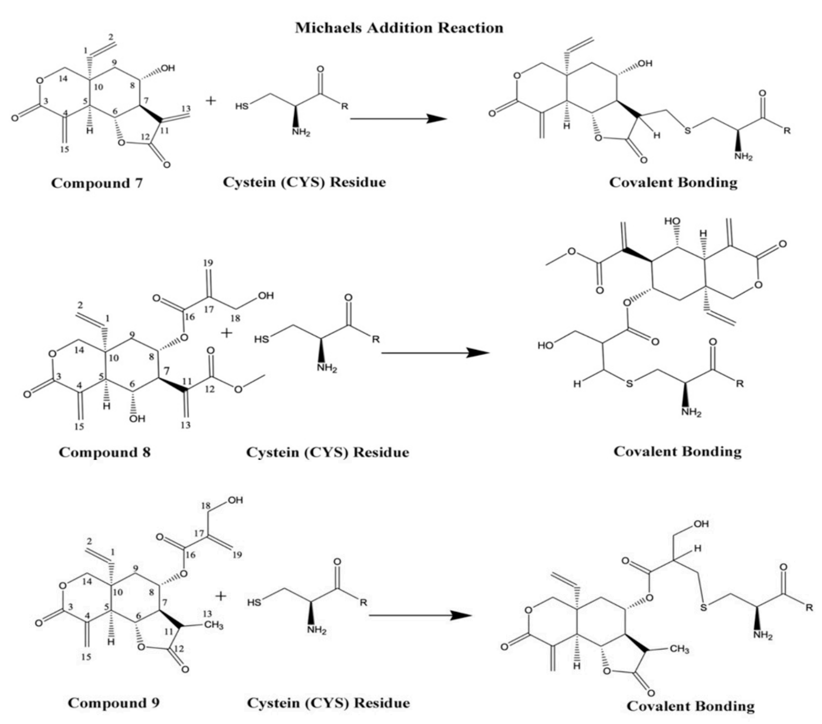

B

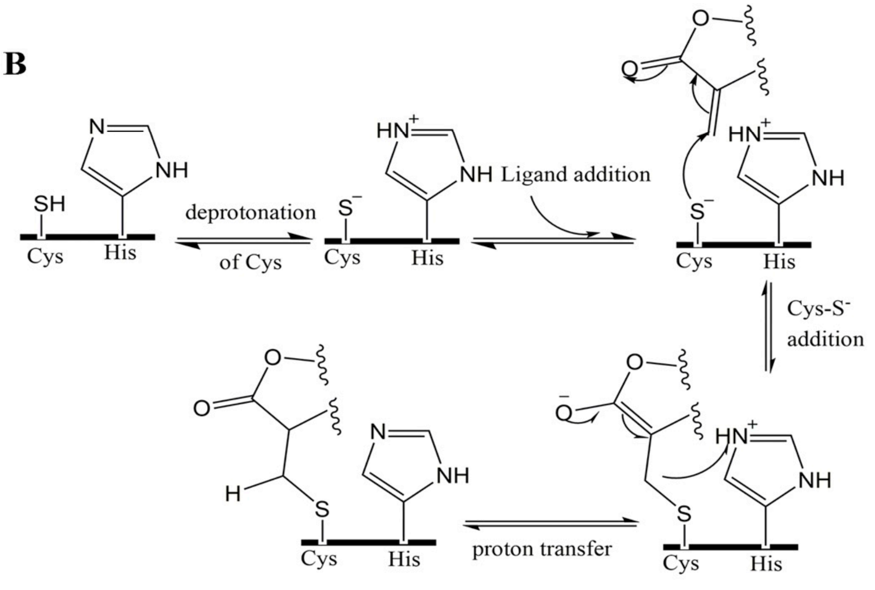

Supplementary Figure 23. (A) Schematic diagram of Michael addition and covalent warheads in compounds **7-9** leading to the devolvement of covalent bond formation with cysteine (CYS) residues in $3CL^{Pro}$ and PL^{Pro}. (B) Mechanistic view of the atomistic and electronic level of the proposed covalent docking between ligands (compounds **7-9**) and CYS145-HIS41 of $3CL^{Pro}$ and CYS111-HIS272 of PL^{Pro}. CYS and HIS in the scheme represent the CYS145-HIS41 of $3CL^{Pro}$ and CYS111-HIS272 of PL^{Pro}.

Supplementary Table 23. Covalent docking protocol resulting auxiliary interactions (hydrogen bond formation) by Vernolepin (compound **7**) with amino acid residues in the active pocket of SARS-CoV-2 target protein ($3CL^{Pro}$).

Ligand (Compound 7)	Receptor ($3CL^{Pro}$)	Interaction Type	Distance (Å)	Energy (kcal/mol)	Docking Score (kcal/mol)
C 17	SD MET 49 (A)	H-donor	3.68	-0.6	-5.3
S 8	N CYS 145 (A)	H-acceptor	2.8	-0.5	
O 33	N GLY 143 (A)	H-acceptor	3.02	-2.2	
C 13	5-ring HIS 41 (A)	H-π	3.65	-0.6	

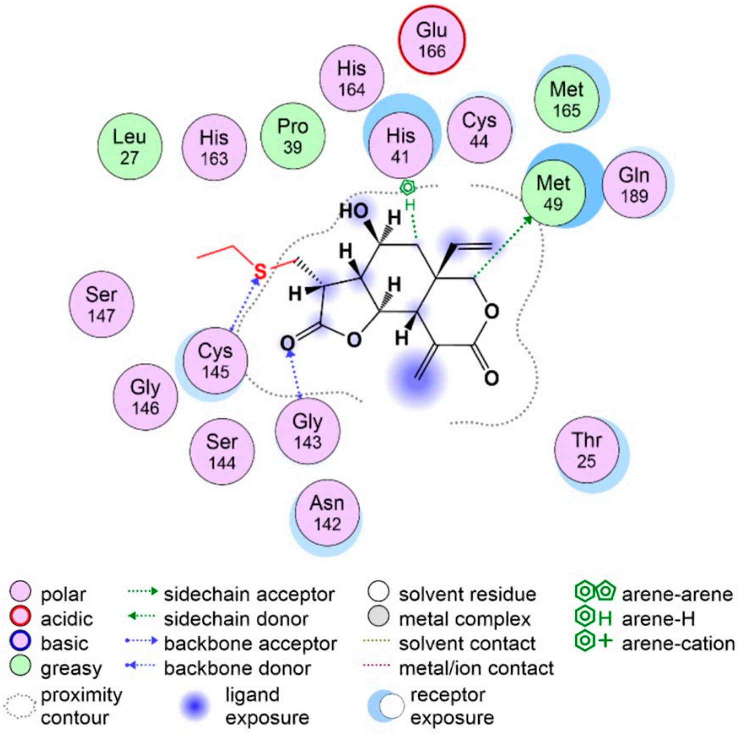

Supplementary Figure 24. 2D view of $3CL^{Pro}$ showing covalent binding with compound **7**. Descriptions of bonds and color in 2D images are shown below the image. Image was generated by using MOE software.

Supplementary Table 24. Covalent docking protocol resulting auxiliary interactions (hydrogen bond formation) by Vernodalol (compound **8**) with amino acid residues in the active pocket of SARS-CoV-2 target protein ($3CL^{Pro}$).

**Ligand (Compound 8)**	**Receptor ($3CL^{Pro}$)**	**Interaction Type**	**Distance (Å)**	**Energy (kcal/mol)**	**Docking Score (kcal/mol)**
O 42	SG CYS 145 (A)	H-donor	3.21	-0.6	
S 8	N CYS 145 (A)	H-acceptor	2.78	-0.5	-6.5
O 42	N GLY 143 (A)	H-acceptor	3.03	-0.6	

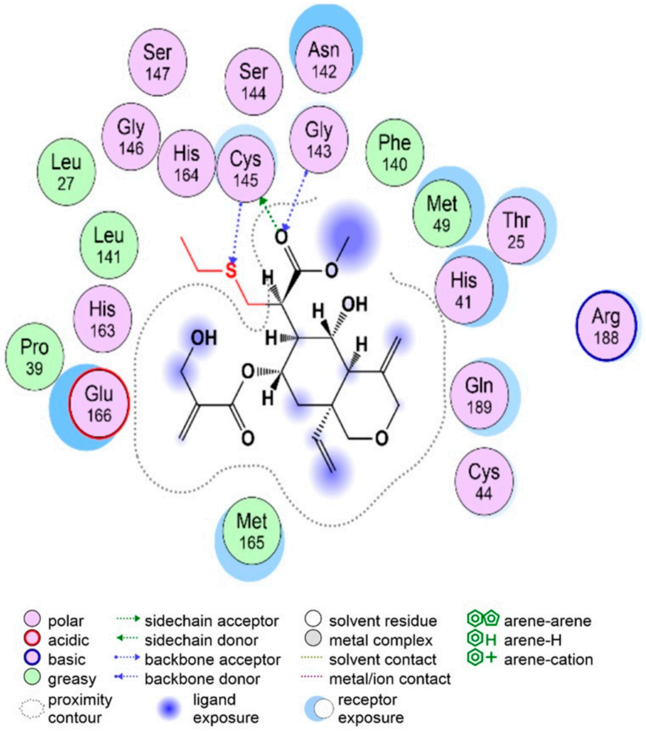

Supplementary Figure 25. 2D view of $3CL^{Pro}$ showing covalent binding with compound **8**. Descriptions of bonds and color in 2D images are shown below the image. Image was generated by using MOE software.

Supplementary Table 25. Covalent docking protocol resulting auxiliary interactions (hydrogen bond formation) by 11β,13-dihydrovernodalin (compound **9**) with amino acid residues in the active pocket of SARS-CoV-2 target protein ($3CL^{Pro}$).

Ligand (Compound 9)	Receptor ($3CL^{Pro}$)	Interaction Type	Distance (Å)	Energy (kcal/mol)	Docking Score (kcal/mol)
C 33	SD MET 49 (A)	H-donor	3.8	-0.4	-6.8
S 8	NE2 HIS 41 (A)	H-acceptor	3.79	-0.8	
C 19	5-ring HIS 41 (A)	H-π	4.08	-1.2	
C 35	5-ring HIS 41 (A)	H-π	4.61	-0.7	

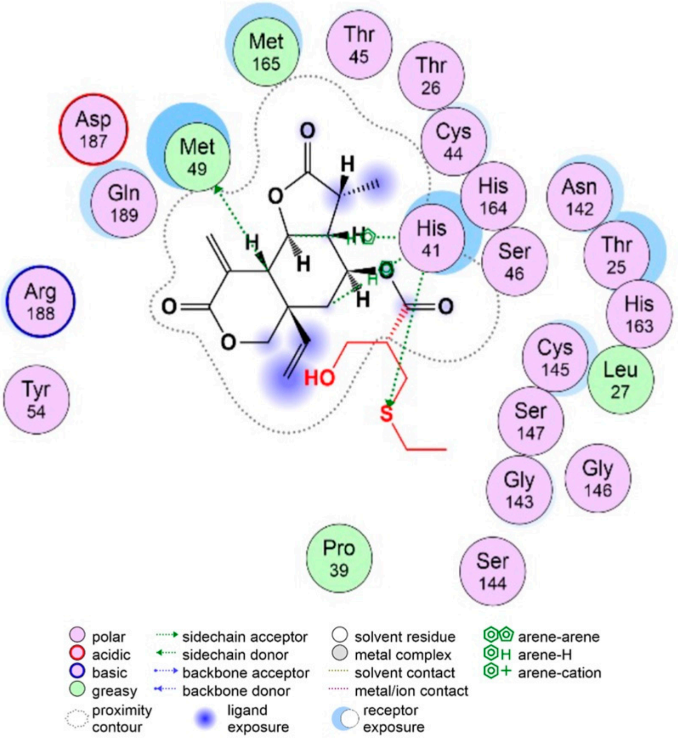

Supplementary Figure 26. 2D view of $3CL^{Pro}$ showing covalent binding with compound **9**. Descriptions of bonds and color in 2D images are shown below the image. Image was generated by using MOE software.

Supplementary Table 26. Covalent docking protocol resulting auxiliary interactions (hydrogen bond formation) by Vernolepin (compound **7**) with amino acid residues in the active pocket of SARS-CoV-2 target protein (PL^{Pro}).

Ligand (Compound 7)	Receptor (PL^{Pro})	Interaction Type	Distance (Å)	Energy (kcal/mol)	Docking Score (kcal/mol)
C 15	SG CYS 270 (B)	H-donor	3.75	-0.5	
S 8	N TYR 273 (B)	H-acceptor	4.07	-2	-4.4
C 37	6-ring TRP 106 (B)	H-π	3.98	-0.5	

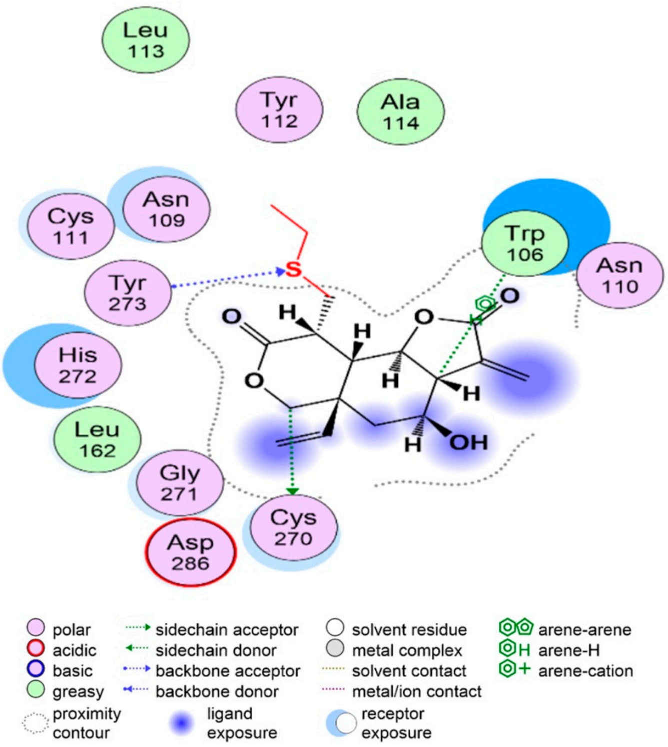

Supplementary Figure 27. 2D view of PL^{Pro} showing covalent binding with compound **7**. Descriptions of bonds and color in 2D images are shown below the image. Image was generated by using MOE software.

Supplementary Table 27. Covalent docking protocol resulting auxiliary interactions (hydrogen bond formation) by Vernodalol (compound **8**) with amino acid residues in the active pocket of SARS-CoV-2 target protein (PL^{Pro}).

Ligand (Compound 8)	Receptor (PL^{Pro})	Interaction Type	Distance (Å)	Energy (kcal/mol)	Docking Score (kcal/mol)
O 57	OD1 ASN 109 (B)	H-donor	2.75	-1.7	-4.9
O 57	SG CYS 270 (B)	H-donor	4.02	-0.7	
S 8	N CYS 111 (B)	H-acceptor	2.87	-0.5	
S 8	N TYR 273 (B)	H-acceptor	4.49	-0.7	
C 25	5-ring TRP 106 (B)	H-π	4.24	-1	
C 31	6-ring TRP 106 (B)	H-π	3.95	-1	

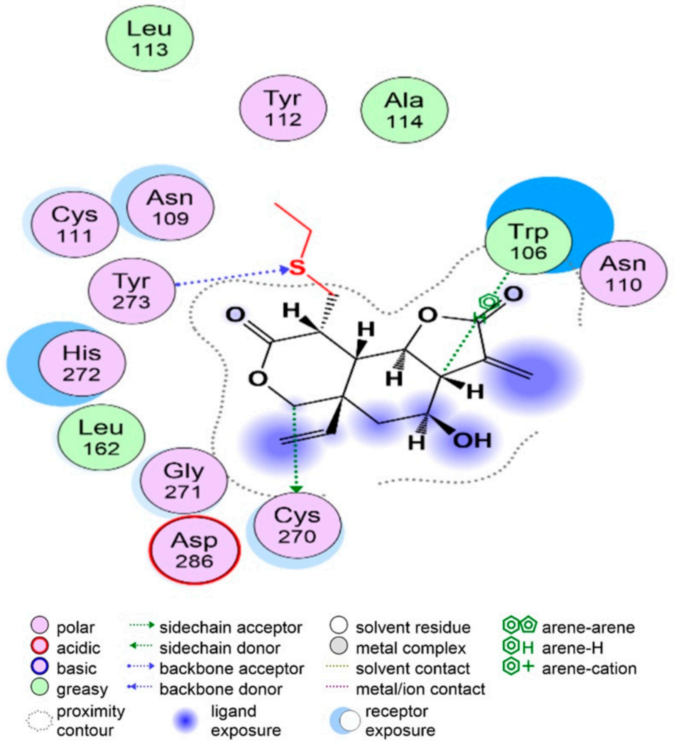

Supplementary Figure 28. 2D view of PL^{Pro} showing covalent binding with compound **8**. Descriptions of bonds and color in 2D images are shown below the image. Image was generated by using MOE software.

Supplementary Table 28. Covalent docking protocol resulting auxiliary interactions (hydrogen bond formation) by 11β,13-dihydrovernodalin (compound **9**) with amino acid residues in the active pocket of SARS-CoV-2 target protein (PL^{Pro}).

Ligand (Compound 9)	Receptor (PL^{Pro})	Interaction Type	Distance (Å)	Energy (kcal/mol)	Docking Score (kcal/mol)
O 14	SG CYS 270 (B)	H-donor	3.77	-0.9	-4.7
C 21	SG CYS 270 (B)	H-donor	3.87	-0.5	
S 8	N TYR 273 (B)	H-acceptor	4.11	-1	
O 15	N CYS 111 (B)	H-acceptor	3.04	-1	

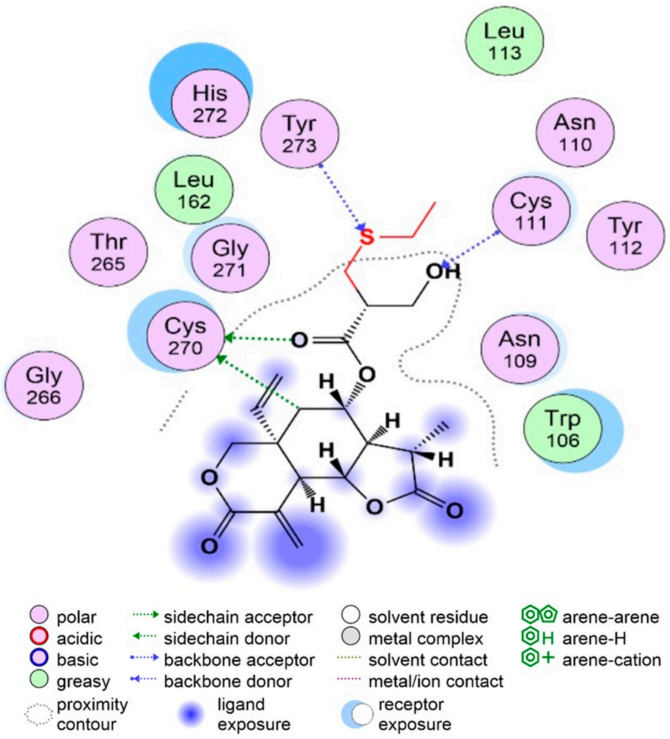

Supplementary Figure 29. 2D view of PL^{Pro} showing covalent binding with compound **9**. Descriptions of bonds and color in 2D images are shown below the image. Image was generated by using MOE software.

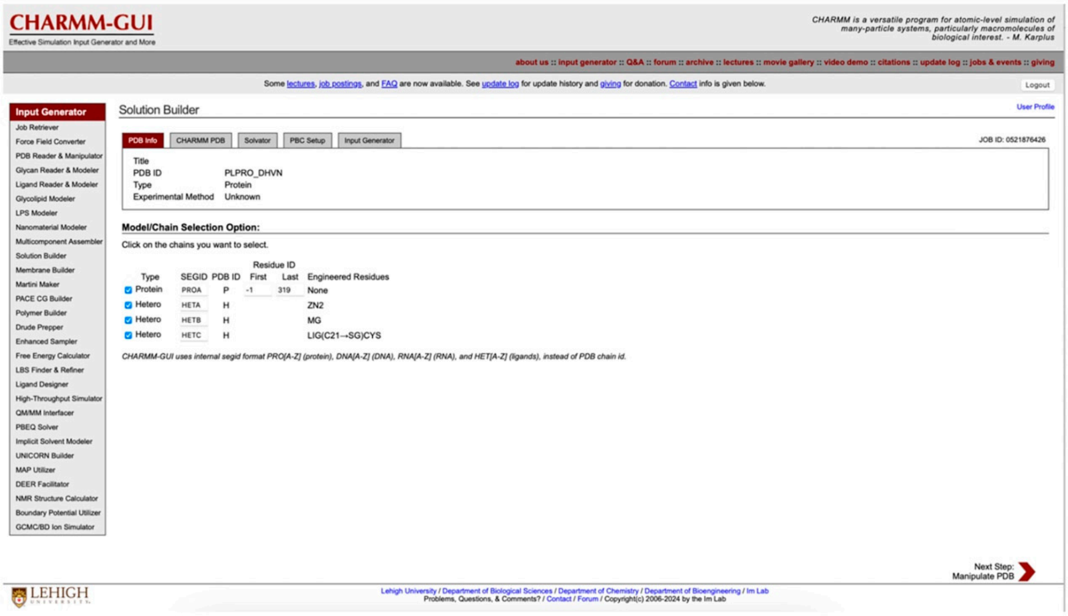

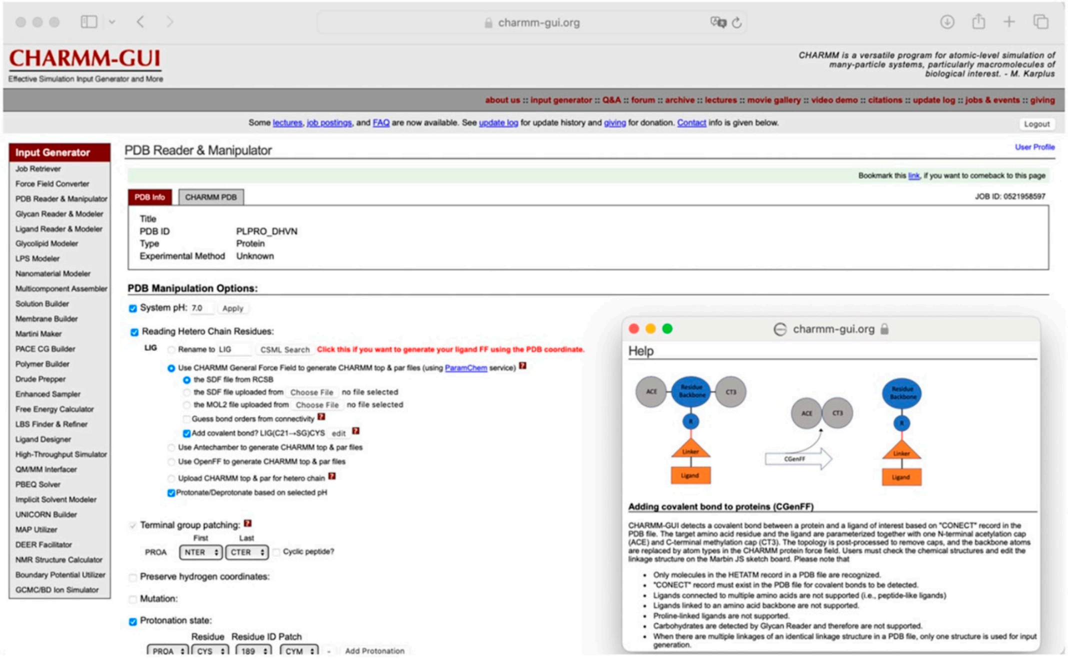

Supplementary Figure 30. Representative screenshots of forcefield parameters in CHARMM-GUI exhibiting the availability of option to upload the molecule covalently bonded with the target, this was implemented in the MD simulation analysis of 3CL^{Pro} and PL^{Pro} covalent bonded with compounds **7**, **8** and **9**.

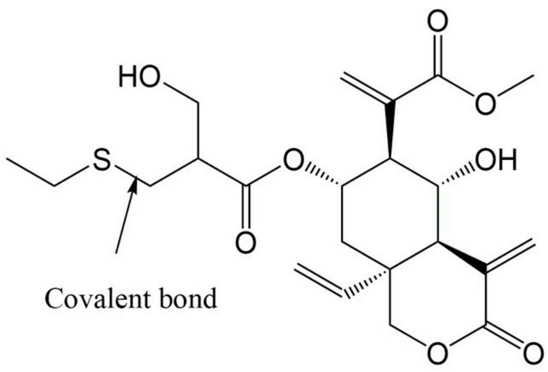

Compound 8

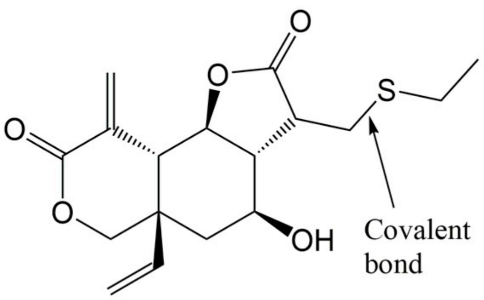

Compound 7

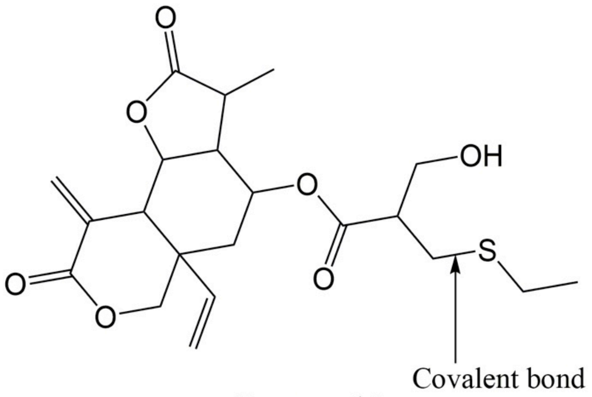

Compound 9

Supplementary Figure 31. Ligands (compounds **7-9**) were edited to add covalent bond with the thiol (SH) of CYS 145/111 in $3CL^{Pro}$ and PL^{Pro} for parameterization. 'Chemical structure of virtual ligand' in ChemAxon online software of CHARMM-GUI was used for above editing.

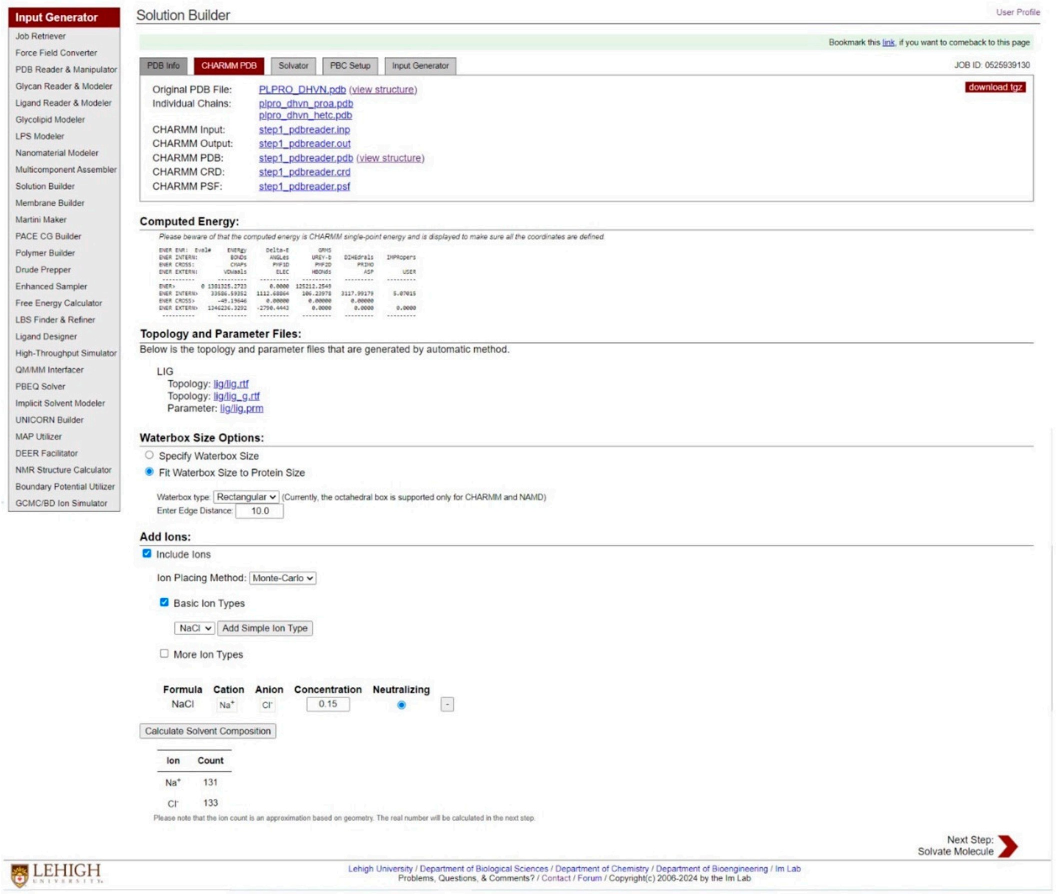

Supplementary Figure 32. Representative screenshot from CHARMM-GUI showing the generation of PDB files that generated the topology and parameter files of the ligands.

Supplementary Table 29. Summarization of interaction between compounds **1-10** resulting in reversible (non-covalent) and irreversible (covalent) binding with $3CL^{Pro}$.

Compound Code	Compound Name	Target ($3CL^{Pro}$)	Binding Mode
**1**	5,3′,4′-trihydroxyflavan 7-O-gallate	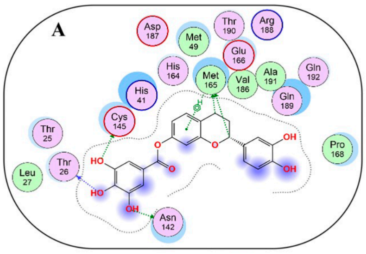 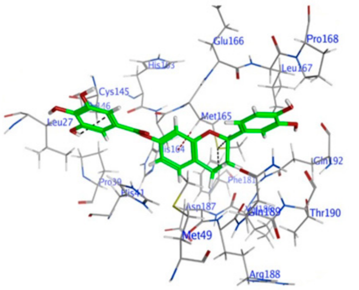	Reversible
**2**	5,4′-dihydroxyflavan 7-3′-O-digallate	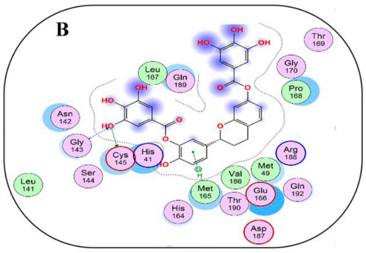 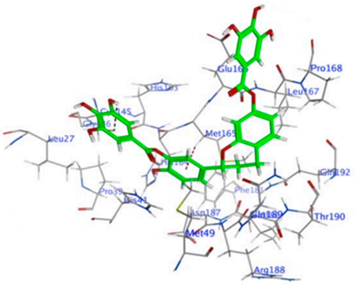	Reversible
**3**	5,3′-dihydroxyflavan 7-4′-*O*-digallate	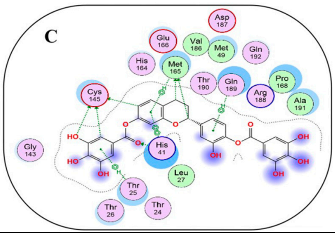	Reversible

		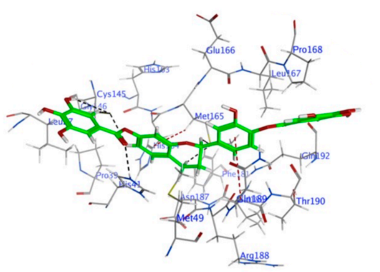	
**4**	Spinasterol	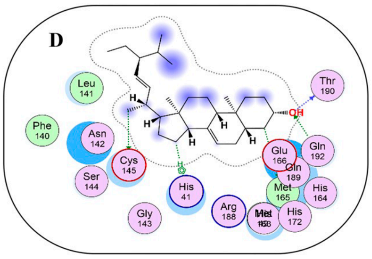 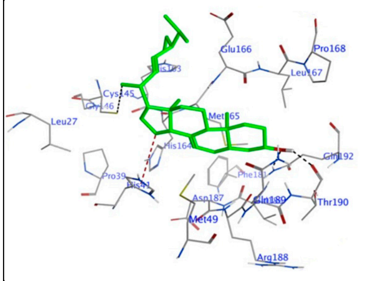	Reversible
**5**	Stigmasterol	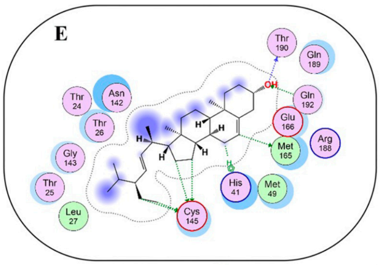 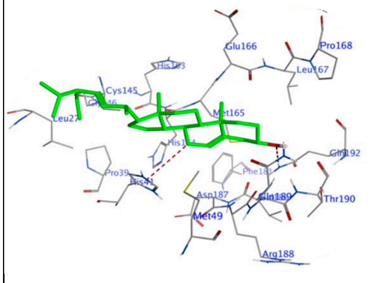	Reversible

**6**	3′,4′,5,7-tetrahydroxy-3-methoxyflavone	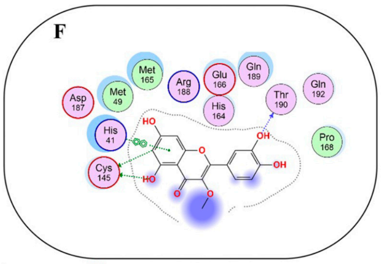<br>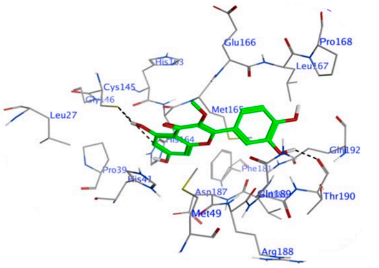	Reversible
**7**	Vernolepin	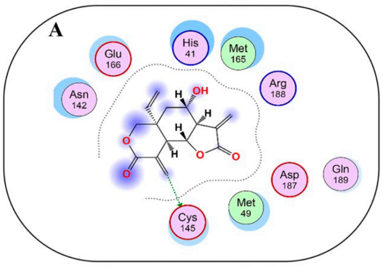<br>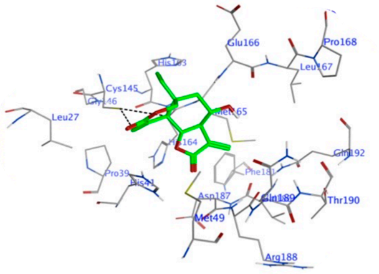	Reversible
		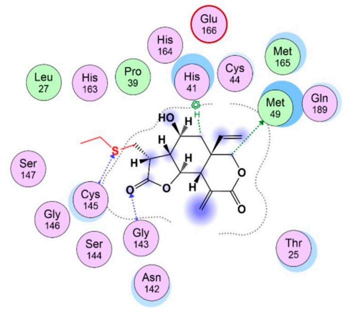	Irreversible

		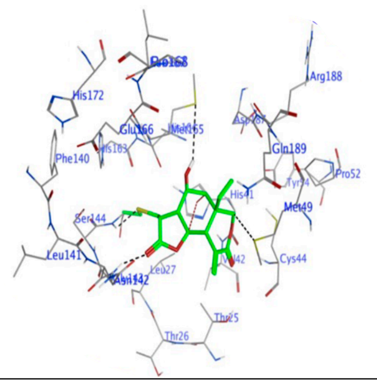	
**8**	Vernodalol	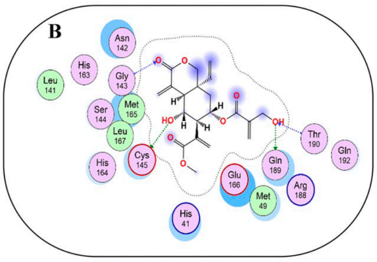 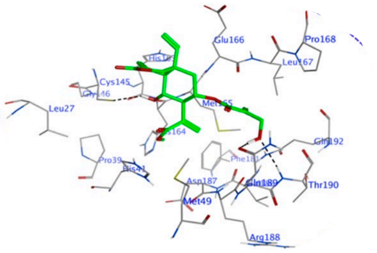	Reversible
		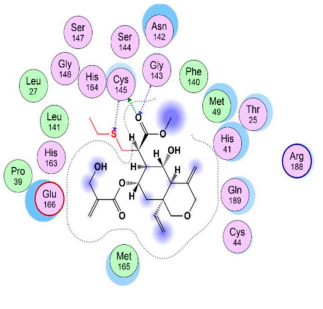	Irreversible

<table>
<tr><td></td><td></td><td>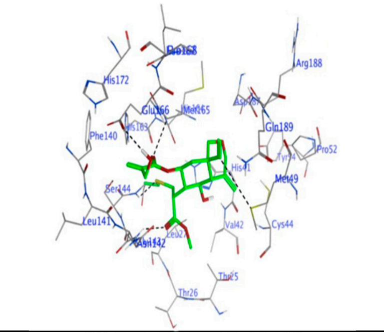</td><td></td></tr>
<tr><td rowspan="2">9</td><td rowspan="2">11β,13-dihydrovernodalin</td><td>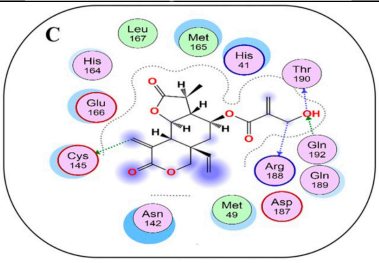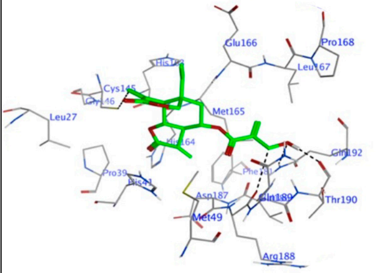</td><td>Reversible</td></tr>
<tr><td>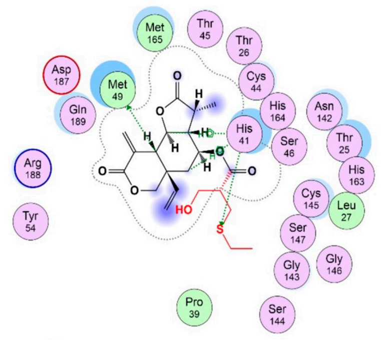</td><td>Irreversible</td></tr>
</table>

		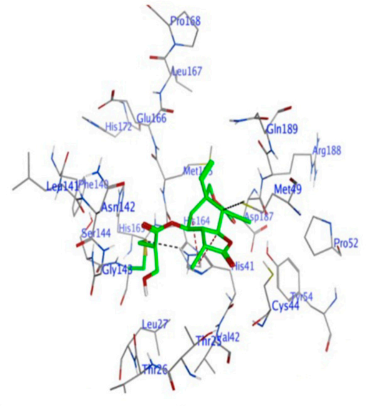	
**10**	Quercitrin<br>3-O-rhamnoside	D<br>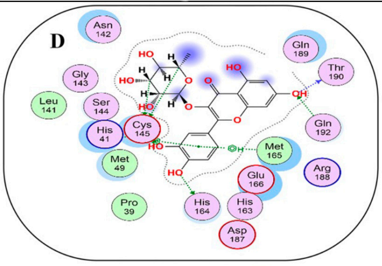<br>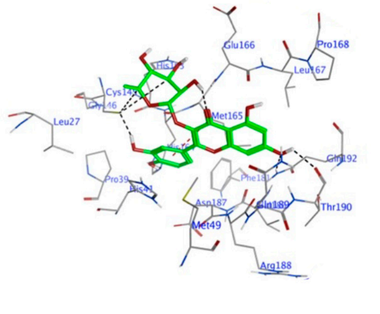	Reversible

The 2D and stick models shown inside the table are the same images that are already described in the manuscript.

Supplementary Table 30. Summarization of interaction between compounds **1-10** resulting in reversible (non-covalent) and irreversible (covalent) binding with PL^{Pro}.

Compound Code	Compound Name	Target (PL^{Pro})	Binding Mode
**1**	5,3′,4′-trihydroxyflavan 7-O-gallate	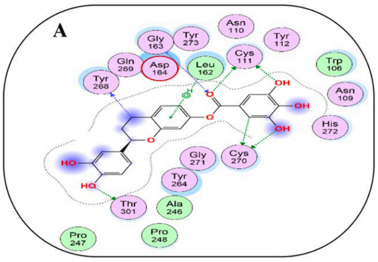<br>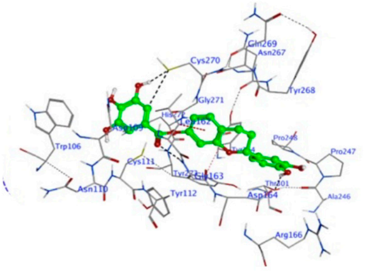	Reversible
**2**	5,4′-dihydroxyflavan 7-3′-O-digallate	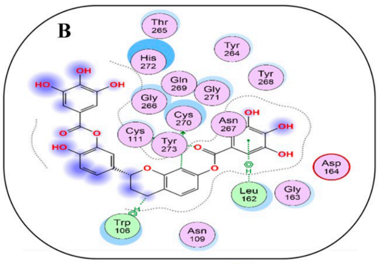<br>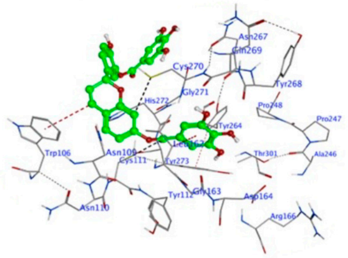	Reversible
**3**	5,3′-dihydroxyflavan 7-4′-*O*-digallate	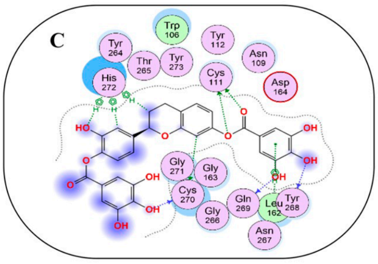	Reversible

		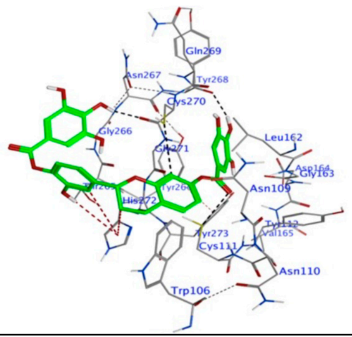	
**4**	Spinasterol	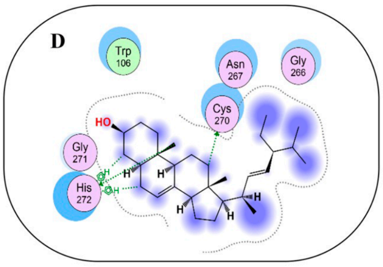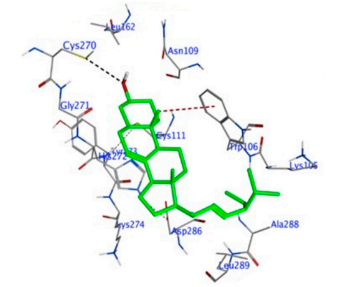	Reversible
**5**	Stigmasterol	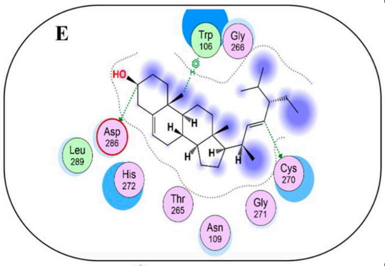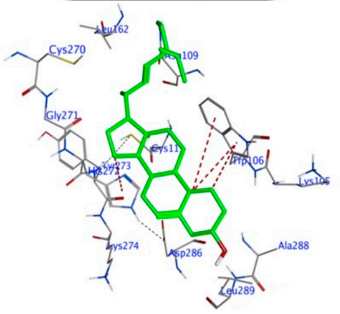	Reversible

<table>
<tr>
<td>6</td>
<td>3′,4′,5,7-tetrahydroxy-3-methoxyflavone</td>
<td>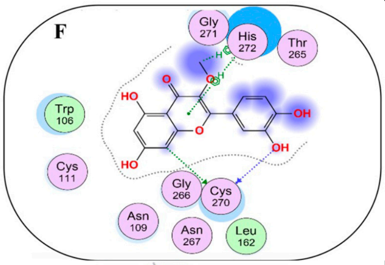

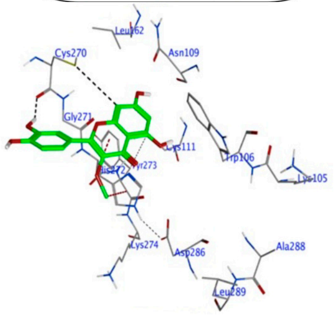
</td>
<td>Reversible</td>
</tr>
<tr>
<td rowspan="2">7</td>
<td rowspan="2">Vernolepin</td>
<td>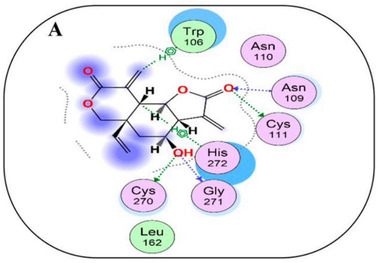

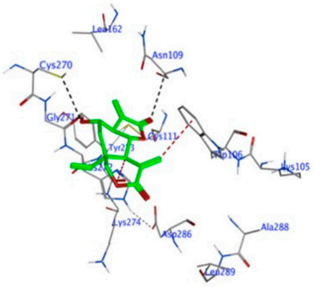
</td>
<td>Reversible</td>
</tr>
<tr>
<td>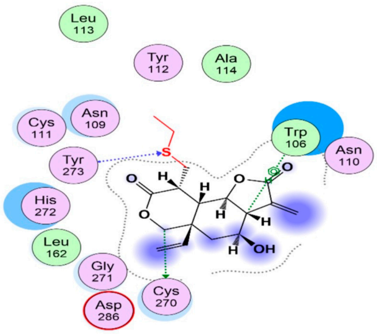
</td>
<td>Irreversible</td>
</tr>
</table>

		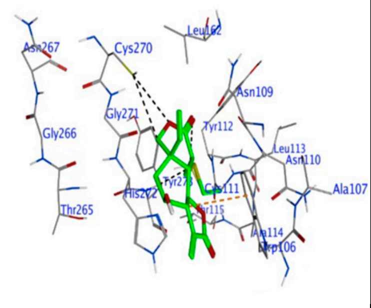	
**8**	Vernodalol	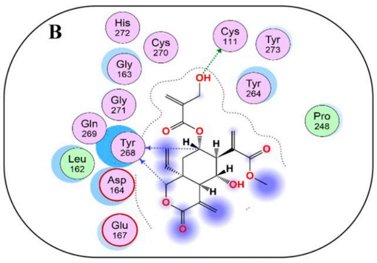 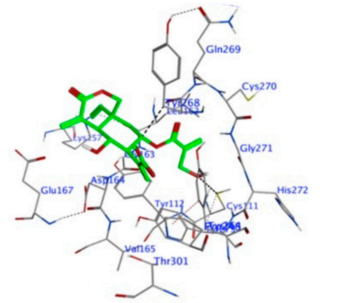	Reversible
		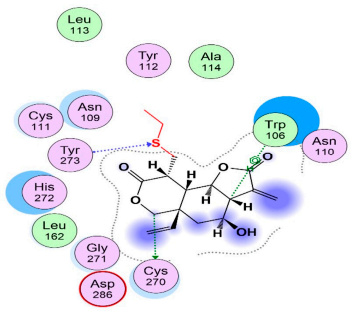	Irreversible

<table>
<tr><td></td><td></td><td>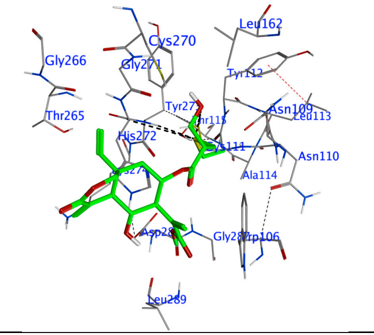
</td><td></td></tr>
<tr><td>9</td><td>11β,13-dihydrovernodalin</td><td>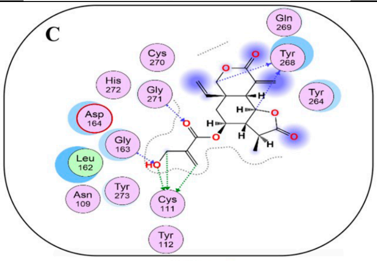

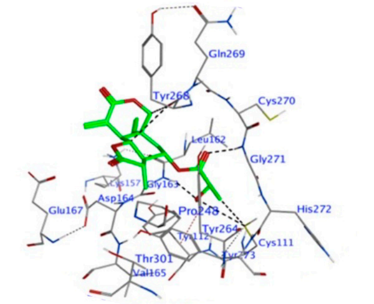
</td><td>Reversible</td></tr>
<tr><td></td><td></td><td>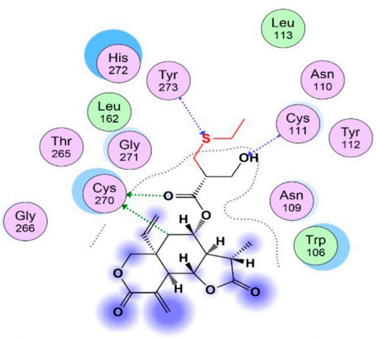

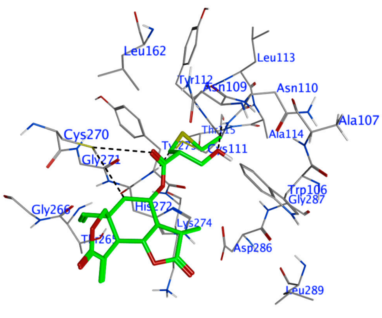
</td><td>Irreversible</td></tr>
</table>

**10**	Quercitrin<br>3-O-rhamnoside	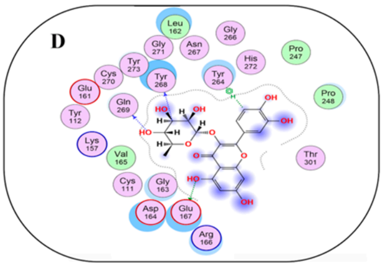<br>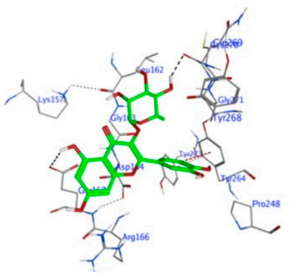	Reversible

The 2D and stick models shown inside the table are the same images that are already described in the manuscript.
